# Targeting ferroptosis opens new avenues for the development of novel therapeutics

**DOI:** 10.1038/s41392-023-01606-1

**Published:** 2023-09-21

**Authors:** Shumin Sun, Jie Shen, Jianwei Jiang, Fudi Wang, Junxia Min

**Affiliations:** The First Affiliated Hospital, Institute of Translational Medicine, The Second Affiliated Hospital, School of Public Health, Cancer Center, State Key Laboratory of Experimental Hematology, Zhejiang University School of Medicine, Hangzhou, China

**Keywords:** Cell biology, Biologics

## Abstract

Ferroptosis is an iron-dependent form of regulated cell death with distinct characteristics, including altered iron homeostasis, reduced defense against oxidative stress, and abnormal lipid peroxidation. Recent studies have provided compelling evidence supporting the notion that ferroptosis plays a key pathogenic role in many diseases such as various cancer types, neurodegenerative disease, diseases involving tissue and/or organ injury, and inflammatory and infectious diseases. Although the precise regulatory networks that underlie ferroptosis are largely unknown, particularly with respect to the initiation and progression of various diseases, ferroptosis is recognized as a bona fide target for the further development of treatment and prevention strategies. Over the past decade, considerable progress has been made in developing pharmacological agonists and antagonists for the treatment of these ferroptosis-related conditions. Here, we provide a detailed overview of our current knowledge regarding ferroptosis, its pathological roles, and its regulation during disease progression. Focusing on the use of chemical tools that target ferroptosis in preclinical studies, we also summarize recent advances in targeting ferroptosis across the growing spectrum of ferroptosis-associated pathogenic conditions. Finally, we discuss new challenges and opportunities for targeting ferroptosis as a potential strategy for treating ferroptosis-related diseases.

## Introduction

Ferroptosis is a unique form of iron-dependent regulated cell death originally identified by screening RSL (RAS-selective lethal) compounds.^1^ The key characteristic of ferroptosis is an extensive accumulation of lipid peroxides,^1^ making ferroptosis genetically, morphologically, and biochemically distinct from other types of cell death. In the decade since ferroptosis was first reported, increasing evidence has emerged suggesting that ferroptosis may play a pivotal role in many biological processes such as tumor suppression and immunity, indicating that ferroptosis is important for maintaining health by regulating metabolism and redox homeostasis. Recently, a plethora of studies have shown that ferroptosis also plays a critical role in a variety of pathophysiological processes such as ischemic organ injury, stroke, cardiac myopathy, and neurodegenerative diseases; ferroptosis has also been implicated in many oncogenic pathways, suggesting it might serve as a target for novel cancer therapeutics.^2^

In mammalian cells, ferroptosis is regulated mainly by iron homeostasis, lipid metabolism, and glutathione-dependent redox balance. Thanks to rapid progress in the study of ferroptosis, numerous efforts have made to identify potent, druggable ferroptosis modulators for use in clinical applications, opening new avenues for developing novel treatment strategies to target many ferroptosis-related diseases, including cancer and heart injury. With respect to cancer, novel ferroptosis agonists have shown promise in several cancer types. On the other hand, antagonists of ferroptosis have been shown to help alleviate ferroptosis-related diseases such as ischemia/reperfusion (I/R)-induced damage, neurodegenerative diseases, and inflammatory diseases.^3–7^

Here, we provide a comprehensive update of recent efforts to target ferroptosis for treating a variety of relevant diseases, and we discuss the safety and efficacy of ferroptosis agonists and antagonists, as well as their pharmacokinetics profiles, their experimental stage in the drug discovery process, and opportunities for further clinical development. We also review the biological roles, molecular mechanisms, and clinical implications of ferroptosis, and we discuss insights into ferroptosis-focused translational research, as well as current knowledge gaps and opportunities. Finally, we discuss future research orientations that will lead to the clinical implementation of these novel ferroptosis modulators for treating a diverse range of diseases.

## Discovery and characteristics of ferroptosis

**Fig. 1:**
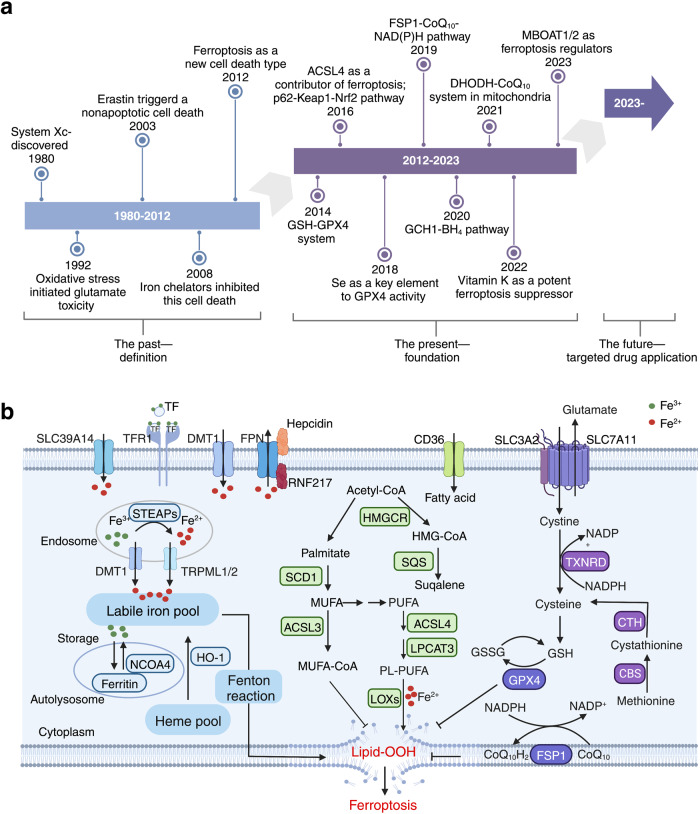
Timeline of the identification and characterization of ferroptosis, and the underlying mechanisms. **a**, Timeline depicting the past, present, and future of ferroptosis. The first time period (1980–2012) ended with the term “ferroptosis” being coined by Dixon et al. in 2012. The present period (2012–2023) developed rapidly, with important details emerging such as the GCH1-BH_4_ and DHODH-CoQ_10_ pathways. The future (2023-?) is expected to bring numerous new applications for targeting ferroptosis. **b**, Three pathways mediate ferroptosis, including iron metabolism, redox, and lipid metabolism. Dysregulation of oxidative-reductive systems, iron metabolism, and/or peroxidation of PUFAs can induce ferroptosis. ACSL4, acyl-CoA synthetase long-chain family member 4; BH_4_, tetrahydrobiopterin; CBS, cystathionine beta-synthase; CD36, cluster differentiation 36; CoQ_10_, coenzyme Q_10_; CTH, cystathionine gamma-lyase; DHODH, dihydroorotate dehydrogenase; DMT1, proton-coupled divalent metal ion transporter 1; FPN, ferroportin; FSP1, ferroptosis suppressor protein 1; GCH1, GTP cyclohydrolase 1; GPX4, glutathione peroxidase 4; GSH, glutathione; GSSG, glutathione disulfide; HO-1, heme oxygenase 1; Keap1, Kelch-like ECH-associated protein 1; LOX, lipoxygenase; LPCAT, lysophosphatidylcholine acyltransferase; MUFA, monounsaturated fatty acid; NCOA4, nuclear receptor coactivator 4; Nrf2, nuclear factor erythroid 2-related factor 2; PL-PUFA, phospholipid-containing polyunsaturated fatty acid; PUFA, polyunsaturated fatty acid; RNF217, E3 ubiquitin protein ligase RNF217; SCD1, stearoyl-coenzyme A desaturase 1; SLC, solute carrier family; SQS, squalene synthase; STEAP, 6-transmembrane epithelial antigen of the prostate metalloreductase family; TF, transferrin; TFR1, transferrin receptor protein 1; TRPML, lysosomal cation channel mucolipin. Created with BioRender.com

Although the term “ferroptosis” was first coined by Dixon et al. in 2012 (Fig. 1a), a similar form of neuronal cell death triggered by cysteine depletion called “oxytosis” was reported by Murphy et al. back in 1989;^8^ this neuronal cell death was induced by the excitotoxin glutamate by inhibiting SLC7A11 (solute carrier family 7 member 11), a component of the cystine-glutamate antiporter system Xc^−^ known to play a role in ferroptosis. Therefore, oxytosis and ferroptosis have been suggested to share several key characteristics, including their gene expression patterns, high activity of lipoxygenases, and high accumulation of reactive oxygen species (ROS).^9^ By 2003, a distinct form of erastin-induced cell death in RAS-expressing cancer cells was receiving considerable attention;^10^ this form of cell death was not inhibited by caspase inhibitors but could be suppressed by treating the cells with iron-chelating agents.^11^ Yang et al. subsequently found that RAS-selective lethal small molecule 3 (RSL3) could also trigger this iron-dependent form of cell death.^12^ Based on these characteristics, Dixon et al. named this type of cell death ferroptosis.^1^ Morphologically, cells undergoing ferroptosis generally have shrunken mitochondria with increased membrane density and reduced—or absent—mitochondrial cristae. Biochemically, ferroptosis is characterized by increased oxidative stress and depleted antioxidative defense. Although ferroptosis is generally well-accepted as a tightly regulated cellular metabolic process, its precise mechanisms such as how excessive phospholipid (PL) peroxidation drives ferroptosis and the tissue- and disease-specific epilipidome signatures remain unknown.

The mechanisms that regulate ferroptosis have begun to emerge (Fig. 1a). For example, Yang et al. found that the glutathione/glutathione peroxidase 4 (GSH/GPX4) pathway serves as an important regulator of ferroptosis.^13^ Soon after, other groups identified ACSL4 (acyl-CoA synthetase long-chain family member 4) as a predominant mediator of ferroptosis.^14,15^ In addition, selenium was shown to protect against ferroptosis.^16^ In 2019, two groups independently reported that the FSP1 (ferroptosis suppressor protein 1)-CoQ_10_ (coenzyme Q_10_)-NAD(P)H pathway inhibits ferroptosis via a GPX4-independent mechanism.^17,18^ In addition, the GCH1 (GTP cyclohydrolase 1)-BH_4_ (tetrahydrobiopterin) pathway was reported as an additional GPX4-independent modulator of ferroptosis.^19^ Recently, Mao et al. identified the DHODH (dihydroorotate dehydrogenase)-CoQ_10_ axis, which is located at the mitochondrial inner membrane, as an important pathway that protects against ferroptosis.^20^ Even more recently, Mishima et al. reported that vitamin K is a potent FSP1-dependent inhibitor of ferroptosis by functionally screening vitamin compounds in *Gpx4*-deficient mouse embryonic fibroblasts.^21^

## Pathways that regulate ferroptosis

A growing body of evidence indicates that ferroptosis is regulated by a complex network involving iron homeostasis, lipid metabolism, and the oxidative-reductive system (Fig. 1b). For example, cellular ferroptosis is driven directly by an accumulation of toxic PL peroxides in membranes, which are produced primarily by the free iron‒induced Fenton reaction and oxidized PL-containing polyunsaturated fatty acids (PL-PUFAs).

Because iron chelators such as deferoxamine (DFO) can block ferroptosis, ferroptosis was originally defined as iron-dependent.^1^ In contrast, iron overload has been shown to sensitize numerous cells types to ferroptosis.^3,22–24^ Therefore, maintaining iron homeostasis is critical in order to protect cells from ferroptosis. The intracellular labile iron pool (LIP) is regulated by the uptake, export, storage, and utilization of iron (Fig. 1b). Cellular iron uptake is regulated primarily by the transferrin/transferrin receptor (TF/TFR) system, which has been shown to play a role in ferroptosis.^25^ Recently, we reported that TF protects against ferroptosis-induced liver damage via SLC39A14 (solute carrier family 39, member 4)-mediated non-TF-bound iron (NTBI).^23^ Excess intracellular iron is sequestered largely by the principal iron-storage protein ferritin (FTH), which protects cells from ferroptosis. Consistent with this protective role, we found that mice lacking Fth in cardiomyocytes develop ferroptosis-induced cardiomyopathy.^24^ Notably, ferritinophagy—an NCOA4 (nuclear receptor coactivator 4)-mediated autophagic degradation of ferritin in the lysosome—has been shown to induce ferroptosis by releasing free iron from ferritin.^26,27^ Moreover, the iron exporter ferroportin (FPN) has also been shown to regulate cellular sensitivity to ferroptosis in vitro.^28^

The metabolism of lipids—particularly PL-PUFAs—critically regulates ferroptosis (Fig. 1b). Using a combination of genome-wide CRISPR screening and microarray analysis of ferroptosis-resistant cell lines, Doll et al. found that the enzyme ACSL4 is a major mediator of ferroptosis.^15^ ACSL4 catalyzes the conversion of free PUFAs to acyl-CoA derivatives (PUFA-CoAs), which can be further catalyzed by lysophosphatidylcholine acyltransferase 3 (LPCAT3) to produce PL-PUFAs. PL-PUFAs participate in the biosynthesis of cellular membranes and are highly susceptible to peroxidation due to their bis-allylic hydrogen atoms. Interestingly, loss of either ACSL4 or LPCAT3 increases cellular insusceptibility to ferroptosis by reducing the production of substrates for PL peroxidation.^15,29,30^ In addition, inhibiting lipoxygenases (LOXs), which are iron-containing enzymes, suppresses erastin-induced ferroptosis via iron- and oxygen-triggered free radical chain reactions, independent of their enzymatic activity.^31^

With respect to oxidative and reductive reactions, several major pathways are involved in protecting against ferroptosis (Fig. 1b). First, the well-characterized system Xc^–^-GSH-GPX4 axis serves as a GPX4-dependent mechanism for scavenging PL peroxides via system Xc^–^-mediated GSH synthesis.^1,13^ Moreover, inhibiting either component of system Xc^–^ (i.e., SLC7A11 or SLC3A2) induces ferroptosis by disrupting cystine uptake, thereby limiting GSH synthesis.^1^ In this pathway, GPX4 serves as an essential regulator of ferroptosis.^13^ Second, in 2019 an FSP1-dependent metabolic pathway was shown to protect against ferroptosis via an GPX4-independent process.^17,18^ Specifically, two groups simultaneously reported that FSP1 traps lipid peroxyl radicals to suppress ferroptosis by reducing CoQ_10_ levels at the plasma membrane,^17,18^ a process that is independent of the system Xc^–^-GSH-GPX4 pathway. Third, Mao et al. showed that mitochondrial inner membrane-located DHODH can protect against ferroptosis by reducing CoQ to form ubiquinol, an antioxidant that inhibits ferroptosis; thus, inhibiting DHODH in cancer cells potently activates ferroptosis in parallel with mitochondrial GPX4, independent of cytosolic GPX4 and FSP1.^20^ Finally, Liang et al. recently identified the PL-modifying enzymes MBOAT1 and MBOAT2 as novel sex hormone‒dependent ferroptosis inhibitors.^32^ Mechanistically, the authors showed that MBOAT1/2 suppress ferroptosis by remodeling cellular PLs to protect cells from ferroptosis independent of GPX4, providing novel ferroptosis-targeted therapeutic strategies to sensitize either estrogen receptor (ER) antagonists in ER-positive breast cancer and androgen receptor (AR) antagonists in AR-positive prostate cancer, respectively.

## The pathological role of ferroptosis in disease

**Fig. 2:**
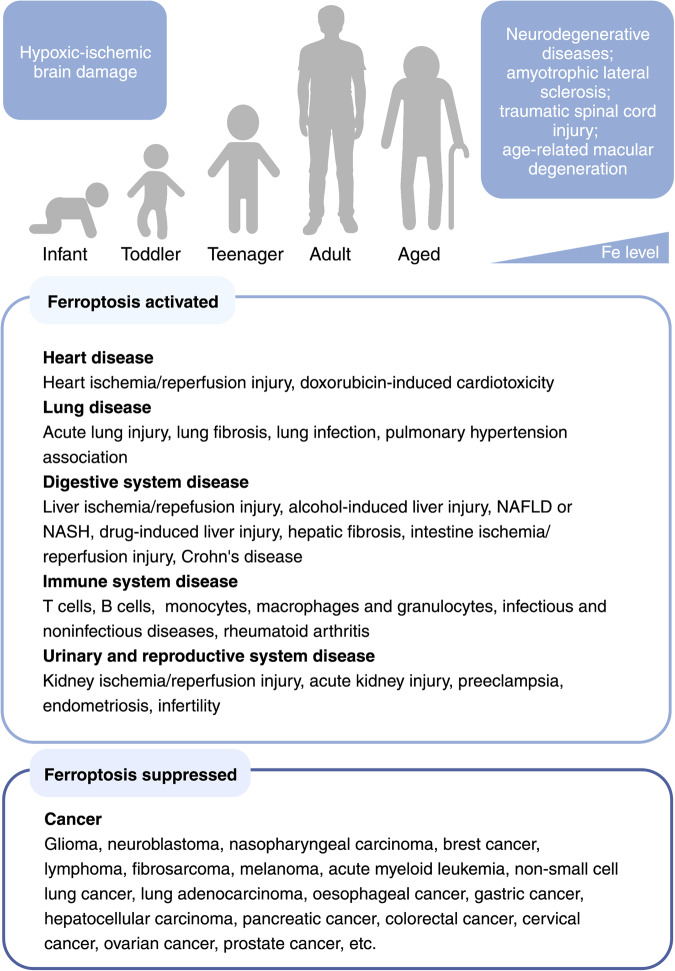
Ferroptosis-related diseases that can present throughout the human lifespan. As we age, iron levels in the body accumulate, inducing ferroptosis and increasing our susceptibility to hypoxic-ischemic brain damage, organ injury‒related diseases, and neurodegenerative diseases. Reduced ferroptosis can cause various forms of cancers in all stages of life. NAFLD non-alcoholic fatty liver disease, NASH non-alcoholic steatohepatitis. Created with BioRender.com

Intracellular accumulation of iron and PL peroxides are two central biochemical events that occur during ferroptosis, and these processes have also been implicated in a wide range of diseases (Tables 1 and 2). Indeed, a plethora of studies suggest a putative causal connection between ferroptosis and the pathophysiological processes underlying many conditions and diseases, including cancer, neurodegeneration, I/R-induced tissue injury, acute kidney damage, and hematologic diseases (Fig. 2).

**Table 1:** Evidence of ferroptosis implicated in human diseases

Type of disease	Disease	Indicator	Method	Refs
Cancers	HCC	GPX4, 4-HNE staining	IHC staining	^215^
	Breast cancer	Serum level of MDA, ferroportin, ferritin; expression of SLC7A11 and SLC3A2 in tumors	Biochemistry; western blot	^348,349^
	Pancreatic adenocarcinoma	Expression of ALOX5 and ALOX12	Real-time PCR; IHC staining	^350^
Neurodegenerative diseases	Alzheimer’s disease	Iron accumulation in hippocampus	MRI	^49,351^
	Parkinson’s disease	Iron deposition in the glia and dopaminergic neurons in the substantia nigra pars compacta	MRI	^52^
	Huntington’s disease	Iron and ferritin accumulation in caudate nucleus, striatum, frontal cortex	MRI; ICP-MS; immunolabeling	^57,352,353^
Tissue and organ injury	COVID-19 induced myocarditis	Oxidized phosphatidylcholine	IHC staining	^66^
	COVID-19 induced renal failure	Oxidized phosphatidylcholine and 4-HNE	IHC staining	^66^
	Hepatic cirrhosis	Decreased TF in serum and liver; iron deposition and MDA staining in liver	Biochemistry; IHC staining	^23^
	NASH	GPX4 expression and GPX4 staining in liver	Western blot; real-time PCR; IHC staining	^354^
	Ulcerative colitis	mRNA expression of FTH1, GPX4, ACSL4, HMOX1, and SLC7A11	Real-time PCR; IHC staining	^355^
	Multiorgan dysfunction syndrome	Catalytic iron and MDA levels in plasma	Biochemistry	^83^
Inflammatory and infectious diseases	Systemic lupus erythematosus	Morphologic changes of mitochondria and lipid-ROS accumulation in neutrophils	TEM; flow cytometry	^88^
	*Pseudomonas aeruginosa* infection	4-HNE	IHC staining	^356^

Abbreviations: *4-HNE* 4-hydroxynonenal; *ACSL4* acyl-CoA synthetase long-chain family member 4; *ALOX* arachidonate lipoxygenase; *FTH1* ferritin heavy chain 1; *GPX4* glutathione peroxidase 4; *ICP-MS* inductively coupled plasma-mass spectrometry; *HCC* hepatocellular carcinoma; *HMOX1* heme oxygenase 1; *IHC* immunohistochemical; *MDA* malondialdehyde; *MRI* magnetic resonance imaging; *NASH* non-alcoholic steatohepatitis; *ROS* reactive oxygen species; *SLC* solute carrier family; *TEM* transmission electron microscopy; *TF* transferrin

**Table 2:** Evidence of ferroptosis in experimental disease models

Type of disease	Disease	Indicator	Method	Refs
Cancer	HCC	Gpx4 and 4-HNE staining	IHC staining	^215^
	Breast cancer	Expression of Slc7a11 and 4-HNE	IHC staining	^357^
	Pancreatic adenocarcinoma	Expression of 4-HNE and Tfr	Western blot; IHC staining IHC staining	^358^
	Neuroblastoma	mRNA expression of Chac1 and Tfr	Real-time PCR	^359^
Neurodegenerative diseases	Alzheimer’s disease	Ultrastructure of neuron mitochondria; Ptgs2, Nrf2, Gpx4, Ho-1, and Nqo1 expression	TEM; western blot	^360^
	Parkinson’s disease	Morphology of mitochondria; Gpx4 and Fth1 expression	TEM; western blot	^361^
	Huntington’s disease	4-HNE adducts in the striatum	Immunofluorescence; confocal microscopy	^56^
Cardiovascular disease	Atherosclerosis	Iron level; Slc7a11 and Gpx4 expression; GSH, NADPH, LPO and MDA levels; Fth, Hmox1 expression	Biochemistry; western blot; real-time PCR; immunofluorescence; IHC staining	^92,362^
	Intracerebral hemorrhagic stroke	MDA level	Immunofluorescence staining	^363^
Tissue and organ injury	Acute kidney injury	GSH and MDA levels; Gpx4 expression	Biochemistry; real-time PCR; western blot	^364^
	Doxorubicin- induced cardiomyopathy	MDA level; Gpx4 expression; 4-HNE staining	Biochemistry; western blot; IHC staining	^3,365^
	I/R-induced cardiomyopathy	Gpx4 expression; 4-HNE staining	Western blot; IHC staining	^3,366^
	Ischemic stroke	Acsl4, Slc7a11 and Gpx4 expression; MDA level	Real-time PCR; biochemistry	^72^
	Alcoholic liver	Lipid peroxides; Slc7a11 and Gpx4 expression	Biochemistry; real-time PCR	^78^
	NASH	Lipid ROS accumulation; morphological of mitochondria; iron accumulation	Biochemistry; TEM	^79^
	Liver fibrosis	4-HNE staining; MDA level	IHC staining; biochemistry	^23^
	Ulcerative colitis	Iron and MDA level, mitochondrial structure; expression of Fth1, Gpx4	Biochemistry; TEM; Western blot	^355^
	Multiorgan dysfunction syndrome	MDA levels in multiple organs	Biochemistry	^83^
Inflammatory and infectious diseases	Systemic lupus erythematosus	Neutrophil viability; lipid-ROS in neutrophil	Flow cytometry	^88^
	*Pseudomonas aeruginosa*	Lipid peroxidation; 4-HNE staining	IHC staining	^356^
	*Mycobacterium tuberculosis*	Gpx4 and Slc7a11 expression; lipid peroxidation	Real-time PCR; flow cytometry	^90^

Abbreviations: *4-HNE* 4-hydroxynonenal; *Acsl4* acyl-CoA synthetase long-chain family member 4; *Chac1* ChaC glutathione specific gamma-glutamylcyclotransferase 1; *Fth* ferritin heavy chain; *Gpx4* glutathione peroxidase 4; *GSH* glutathione; *HCC* hepatocellular carcinoma; *Hmox1* Heme-oxygenase 1; *IHC* immunohistochemical; I/R: ischemia/reperfusion; *LPO* lipid peroxide; *MDA* malondialdehyde; *NASH* non-alcoholic steatohepatitis; *Nqo1* quinone oxidoreductase-1; *Nrf2* nuclear factor erythroid 2-related factor 2; *Ptgs2* prostaglandin-endoperoxide synthase 2; *ROS* reactive oxygen species; *Slc* solute carrier family; *TEM* transmission electron microscope; *Tfr* transferrin receptor

### Cancer

Apoptosis was long considered the principal form of cell death in various cancer types; however, apoptosis-based cancer therapies have limited clinical benefit.^33^ Therefore, identifying novel cancer therapeutics that target pathways other than apoptosis is now an urgent unmet clinical need. Indeed, the notion of ferroptosis originated from lethal screening of compounds in *RAS*-mutated cancer cells.^1^ In support of this notion, ferroptosis has been functionally validated in cancer types with increased PL peroxidation, including hepatocellular carcinoma (HCC),^34^ pancreatic ductal adenocarcinoma,^35^ triple-negative breast cancer (TNBC),^36,37^ and renal cell carcinoma.^38,39^ Thus, these specific cancer types may be more sensitive to ferroptosis-inducing compounds.

It is important to note that ferroptosis has also been closely linked to resistance to various cancer treatments. For example, several studies found that cancer cells with a high-mesenchymal state are more resistant to a variety of cancer treatments, but are particularly susceptible to ferroptosis-inducing compounds.^15,40^ In addition, priming cancer cells with ferroptosis-inducing compounds can sensitize the cancer cells to subsequent immunotherapies.^41^ Taken together, these findings highlight the promise of targeting ferroptosis as a novel strategy for treating various forms of cancer.

### Neurodegenerative diseases

Iron deposition and lipid peroxidation are common pathophysiological features in various neurological diseases. Notably, the disease group known as neurodegeneration with brain iron accumulation (NBIA) refers to neurodegenerative disorders associated with altered iron metabolism in the lesion area and includes Alzheimer’s disease (AD),^42^ Parkinson’s disease (PD),^43^ and Huntington’s disease (HD).^44^ In addition, ablating or inactivating GPX4 promotes neuronal damage and neurodegeneration.^6,7^ In experimental models of degenerative brain conditions, both ferroptosis inhibitors and iron chelators were shown to improve outcome and prognosis.^45–48^

With respect to AD, several studies have shown that dysregulated iron metabolism is linked to ROS production, mitochondrial dysfunction, and neurodegeneration.^42^ Iron deposition in the brain, accompanied by a decrease in endogenous antioxidant capacity, has been associated with the pathogenesis of AD, and iron levels in the brain have been correlated with disease progression.^49^ Furthermore, accumulated iron interacts with the Aβ peptide and tau protein via the formation of a peptide-hemin complex, possibly implicating ferroptosis.^50^

PD is characterized by reduced dopaminergic neurons selectively in the substantia nigra and the presence of Lewy bodies.^51^ In the substantia nigra pars compacta of affected patients, iron was shown to accumulate in glia cells and dopaminergic neurons, with the level of iron correlated with disease severity.^52^ Moreover, reports suggest that the increased oxidative stress present in PD may be attributed to decreased GSH levels in the substantia nigra.^53^ In addition, the brain consumes high amounts of oxygen, making this organ more sensitive to lipid peroxidation.^54^

HD, a hereditary neurodegenerative disease due to an abnormally high number of CAG repeats in the huntingtin (*HTT*) gene,^55^ has also been linked to ferroptosis in animal models.^56^ Excess iron remains a major cause of oxidative stress in neurons, which directly induces ferroptosis during the pathogenesis of HD.^57^ Interestingly, similar to PD, reduced levels of GSH have also been observed in HD.^58^ Together, these findings suggest that ferroptosis may be involved in the pathogenesis of HD.

### Tissue and organ injury

Ferroptosis was originally identified as the major pathogenic driver in several conditions related to organ injury. In 2014, mice lacking *Gpx4* were shown to develop spontaneous acute renal failure and hepatic I/R-induced damage via ferroptosis.^5^ Subsequently, the pathogenic role of ferroptosis in the progression of I/R-induced acute damage in the lungs, heart, and intestinal tract has been validated in animal models.^3,4,59^ We previously systematically reviewed the varied role of ferroptosis in liver disease, and we refer the reader to this review.^60^ Notably, tissue-specific *Gpx4* knockout mice present with tissue damage accompanied by massive death of photoreceptor cells^61^ and endothelial cells.^62^

Acute kidney injury (AKI) refers to sudden-onset kidney failure and/or kidney damage characterized by a rapid loss of the kidney’s excretory function caused by massive levels of cell death and inflammation.^63^ In mouse kidney tubular cells, Gpx4 has been reported to prevent AKI by blocking ferroptosis.^5^ Around the same time, Linkermann et al. independently found that ferroptosis plays a critical role in synchronizing kidney tubular cell death in both severe I/R-induced injury and oxalate crystal‒induced models of AKI; moreover, the authors showed that SRS 16–86—a novel third-generation ferroptosis-specific inhibitor—potently protected against AKI.^64^ Recently, Li et al. reported that ferroptosis is the primary form of cell death causing folic acid‒induced AKI in mice.^65^

In the heart, we first identified ferroptosis as the major pathogenic mechanism in both doxorubicin- and I/R-induced cardiomyopathy, and we showed that targeting ferroptosis significantly alleviated heart injury in mouse models.^3^ In addition, inhibiting glutaminolysis protected against I/R-induced ferroptotic heart damage in vitro, indicating that glutaminolysis plays an essential role in regulating ferroptosis during heart injury.^25^ Recently, Jacobs et al. suggested the possible presence of ferroptosis in the myocardium of a patient with COVID-19‒induced myocarditis.^66^ Using the antibody E06 to stain oxidized phosphatidylcholine, they found that a ferroptosis “signature” may be specific to injured cardiomyocytes in COVID-19‒induced myocarditis, as this signature was not present in either myocarditis of unknown etiology or in COVID-19 patients who presented without myocarditis.^66^ Recently, we summarized the role of ferroptosis in regulating cardiovascular disease, and we refer to the reader to this review.^67^

Previous studies using animal models of brain injury showed features resembling ferroptosis, including increased lipid peroxidation, increased intracellular iron levels, and decreased GSH levels.^68,69^ A subsequent study also showed that inhibiting ferroptosis prevented the death of primary oligodendrocytes.^70^ Notably, organotypic hippocampal slice cultures with ferroptosis-specific inhibitors were shown to prevent neuronal death and decrease hemoglobin-induced iron accumulation, suggesting a pathogenic role of ferroptosis in intracerebral hemorrhage.^71^ In addition, neurons obtained from an ischemic stroke mouse model were shown to have significantly decreased levels of GSH^72^ and increased lipid peroxidation,^73^ suggesting neuronal ferroptosis.^74^ In addition, carvacrol (the major monoterpenic phenol found in some essential oils and known to reduce oxidative stress and apoptosis) has been shown to help protect hippocampal neurons from ferroptosis by upregulating *Gpx4* expression in a gerbil model of cerebral ischemia.^75^ In spinal cord injury, recent studies suggest that ferroptosis contributes to secondary injury, and blocking ferroptosis may help repair traumatic spinal cord injury.^76^

The liver serves as a central metabolic organ and is highly susceptible to various toxic metabolites. We previously showed that high dietary iron directly leads to ferroptosis-induced liver injury, and this damage was aggravated by either knocking out *Slc7a11* expression^77^ or increasing non-TF-bound iron (NTBI).^23^ Ferroptosis was recently linked to various chemical-induced forms of liver injury such as alcoholic liver disease,^78^ methionine/choline-deficient‒induced non-alcoholic steatohepatitis (NASH),^79^ and arsenic-induced NASH.^80^ In addition, both ferroptosis and a concomitant accumulation of lipid ROS were also observed in CCl_4_-induced liver fibrosis,^23^ acetaminophen-induced liver damage,^81^ and I/R-induced liver injury.^82^

Critically ill patients with multiorgan dysfunction syndrome (MODS) were shown to present with a ferroptosis signature that includes increased plasma levels of MDA (malondialdehyde, a lipid peroxidation degradation product) and catalytic iron.^83^ In addition, a ferroptosis signature was shown in tissue samples obtained from a patient with COVID-19‒induced MODS (including cardiac and renal failure).^66^ Using an experimental model of MODS, Van Coillie et al. showed that excess iron can trigger ferroptosis in multiple organs; moreover, they showed that the novel ferroptosis inhibitor UAMC-3203 may be a viable therapeutic agent for the clinical treatment of MODS.^83^

### Inflammatory and infectious diseases

Ferroptosis has also been reported to directly modulate the host’s immune response and inflammation during inflammatory and infectious diseases.^84^ Moreover, the functions of various immune cell types such as T cells, B cells, and macrophages can be affected by ferroptosis. For example, Gpx4-deficient CD4^+^ and CD8^+^ T cells undergo ferroptosis due to accumulated lipid peroxides.^85^ Thus, these ferroptotic T cells are unable to proliferate and have reduced function against the acute lymphoblastic choriomeningitis virus and Leishmania major parasites, suggesting that Gpx4 is required for maintaining T cell‒mediated immunity by protecting the cells against ferroptosis.^85^ With respect to B cells, various B cell populations have been shown to respond differently to ferroptosis induction.^86^ Similarly, in bone marrow cells inducible nitric oxide synthase (iNOS) is more abundant in M1 macrophages than in M2 macrophages, and M1 macrophages tend to be more resistant to iron deposits and ferroptosis.^87^ Interestingly, a recent study found that Gpx4-dependent ferroptosis in neutrophils drives the pathogenesis of systemic lupus erythematosus (SLE),^88^ suggesting that targeting ferroptosis-induced neutropenia may be a potential strategy for treating autoimmune diseases such as SLE.

The aerobic gram-negative bacterium *Pseudomonas aeruginosa* can infect both immunocompetent and immunocompromised hosts. Interestingly, this pathogen expresses lipoxygenase (which oxidizes AA-PE to 15-hydroperoxide AA-PE in host cells) and drives ferroptosis in human bronchial epithelial cells, as well as in clinically isolated cells from patients with persistent lower respiratory tract infection.^89^ Thus, targeting ferroptosis may be a viable strategy for managing *P. aeruginosa* infection.

The bacterium *Mycobacterium tuberculosis* is one of the major pathogens that causes tuberculosis, an infectious disease that affects the lungs, bone tissue, brain, and spine. Interestingly, *M. tuberculosis* has been shown to trigger ferroptosis in macrophages, accompanied by decreased GPX4 expression, increased iron content, and elevated levels of membrane lipid peroxidation.^90^ Similar results were also obtained in mice acutely infected with *M. tuberculosis*.^90^ Given these findings, ferroptosis may also be a promising therapeutic target in patients with tuberculosis and other infectious diseases.

Notably, ferroptosis has also been closely linked to sterile inflammation, a process that occurs in the absence of pathogens and is commonly triggered by release of the intracellular contents from damaged and/or necrotic cells. Sterile inflammation is often triggered by acute conditions such as I/R-induced injury, trauma, oxalate crystal‒induced inflammation, and chronic inflammatory diseases such as atherosclerosis. For instance, ferroptosis increases the recruitment of neutrophils to damaged heart tissue under ischemic conditions, and ferroptosis-specific inhibitors can alleviate this inflammatory damage.^91^ In addition, blocking ferroptosis was shown to significantly reduce atherosclerosis in *ApoE*^*−/−*^ mice fed a high-fat diet.^92^

## Pharmacological agents that target ferroptosis-regulating pathways

Given that iron metabolism, oxidative-reductive pathways, and lipid metabolism coordinately control ferroptosis, altering these three pathways using genetic and pharmacological approaches has been shown to affect ferroptosis (Table 3). Below, we summarize the bioactive pharmacological agents that affect ferroptosis by targeting core components in these three ferroptosis-regulating processes (see also Table 4).

**Table 3:** Genetic and pharmacologic modulators of ferroptosis

Method/Effector	Target	Effect on ferroptosis	Refs
**Genetic interventions**			
Knockdown	*TFR1*	Inhibition	^25^
Knockdown	*IREB2*	Inhibition	^1^
Knockdown	*NCOA4*	Inhibition	^27^
Overexpression	*GCH1*	Inhibition	^19^
Overexpression	*GPX4*	Inhibition	^13^
Knockdown	*KEAP1*	Inhibition	^367^
Knockdown	*POR*	Inhibition	^263^
Overexpression	*SLC7A11*	Inhibition	^1^
Knockdown	*ACSF2*	Inhibition	^1^
Knockout	*ACSL4*	Inhibition	^15,30^
Knockdown	*ALOXs*	Inhibition	^31^
Knockdown	*LPCAT3*	Inhibition	^30^
Knockout	*SQLE*	Inhibition	^368^
Knockdown	*SQS*	Inhibition	^212^
Knockout	*FTH1*	Activation	^24^
Knockout	*DHODH*	Activation	^20^
Knockdown	*GCH1*	Activation	^19^
Knockdown	*GPX4*	Activation	^5,13^
Overexpression	*KEAP1*	Activation	^369^
Knockdown	*SLC7A11*	Activation	^1^
*p53*^*3KR*^ mutation	*SLC7A11*	Activation	^239^
Knockdown	*NQO1*	Activation	^34^
Knockdown	*NRF2*	Activation	^34^
Knockout	*FSP1*	Activation	^370^
**Pharmacological approaches**			
Iron chelators	Iron abundance	Inhibition	^1^
β-ME	GSH synthesis	Inhibition	^1^
Glutathione	GSH synthesis	Inhibition	^1^
NAC	GSH synthesis	Inhibition	^1^
Compound 1d4	GPX4 activity	Inhibition	^219^
Selenium	GPX4 activity	Inhibition	^16^
Fer-1	Lipid ROS	Inhibition	^1^
Lip-1	Lipid ROS	Inhibition	^5^
UAMCs	Lipid ROS	Inhibition	^83,264^
RTAs	Lipid ROS	Inhibition	^1,5,17,18,371^
SSO	CD36	Inhibition	^278^
Thiazolidinediones	ACSL4	Inhibition	^15^
Zileuton	ALOXs	Inhibition	^31,254^
FAC	Iron abundance	Activation	^1^
FINO_2_	Iron oxidation	Activation	^214^
Brequinar	DHODH	Activation	^20^
BSO	GCL	Activation	^13^
Erastin and analogs	System Xc^–^	Activation	^1,13,152,175^
SAS	System Xc^–^	Activation	^1^
RSL3	GPX4	Activation	^1^
ML162	GPX4	Activation	^13,209^
ML210	GPX4	Activation	^211^
iFSP1	FSP1	Activation	^17^
NPD4928	FSP1	Activation	^231^
Trigonelline	NRF2	Activation	^34^
Auranofin	TXNRD1	Activation	^22^
FIN56	SQS	Activation	^212^
Statins	HMGCR	Activation	^40,212^

Abbreviations: *ACSF2* acyl-CoA synthetase family member 2, *ACSL4* acyl-CoA synthetase long-chain family member 4, *ALOX* arachidonate lipoxygenase, *BSO* buthionine sulphoximine, *CD36* cluster differentiation 36, *DHODH* dihydroorotate dehydrogenase, *FAC* ferric ammonium citrate, *Fer-1* ferrostatin-1, *FSP1* ferroptosis suppressor protein 1, *Fth1* ferritin heavy chain 1, *GCH1* GTP cyclohydrolase 1, *GCL* glutamate-cysteine ligase, *GPX4*, glutathione peroxidase 4, *GSH* glutathione, *HMGCR* 3-hydroxy-3-methylglutaryl-CoA reductase, *IREB2* iron response element-binding protein 2, *KEAP1* Kelch-like ECH-associated protein 1, *Lip-1* Liproxstatin-1, *LPCAT3* lysophosphatidylcholine acyltransferase 3, *β-ME* β-mercaptoethanol, *NAC* *N*-acetylcysteine, *NCOA4* nuclear receptor coactivator 4, *NQO1* quinone oxidoreductase-1, *NRF2* nuclear factor erythroid 2-related factor 2, *POR* cytochrome p450 oxidoreductase, *ROS* reactive oxygen species, *RSL3* RAS-selective lethal small molecule 3, *RTA* radical trap antioxidant, *SAS* sulfasalazine, *SLC7A11* solute carrier family 7 member 11, *SQLE* squalene epoxidase, *SQS* squalene synthase, *SSO* sulfosuccinimidyl oleate, *TFR1* transferrin receptor 1, *TXNRD1* thioredoxin reductase 1

**Table 4:** Summary of small molecules that target ferroptosis in vivo and in clinical trials

Targeted pathway	Agent	Effect on ferroptosis	Proposed mechanism	Disease model	Refs	Indication	NCT #	Phase	Status
Iron metabolism	Compound 9a	Inhibitor	Perturbation of NCOA4-Fth1 interaction	Ischemic stroke MCAO rats	^136^	N/A	N/A	N/A	N/A
	CPX	Inhibitor	Iron chelator	MDAY-D2 murine leukemia cells metastasis in NOD/SCID mice; OCI-AML2 and K562 cells xenografts in NOD/SCID mice; polycystic kidney disease model with *Pkd1*^*RC/RC*^ *Pkd2*^*+/–*^	^121,372^	Relapsed or refractory hematologic malignancy	NCT00990587	I	Completed
						Vulvar Cancer	NCT00382330	N/A	Withdrawn
	DFO	Inhibitor	Iron chelator	MCD-induced NASH in mice; hepatic I/R injury in mice; aged (15–18 months) C57 mice exposed to LPS	^82,109,373^	Hypotension, acute renal failure	NCT00870883	II	Completed
						Ischemic stroke	NCT00777140	II	Completed
						AKI	NCT04633889	II	Recruiting
						Aneurysmal subarachnoid hemorrhage	NCT04566991	II	Recruiting
	DFP	Inhibitor	Iron chelator	DSS-induced ulcerative colitis in mice	^374^	Acute myocardial infarction Type 1	NCT05604131	I	Recruiting
						Neurodegeneration with brain iron accumulation (NBIA)	NCT00907283	II	Active, not recruiting
						Stroke	NCT05111821	II	Recruiting
	DFX	Inhibitor	Iron chelator	ICH mice; mid-thoracic spinal contusion rat	^116,118^	Myelodysplasia	NCT03387475	II	Recruiting
						Sickle cell disease	NCT05392101	II	Recruiting
	DXZ	Inhibitor	Iron chelator	DOX- and I/R-induced cardiomyopathy in mice	^3^	During congenital heart surgery	NCT04997291	I	Recruiting
						Preventing heart-related side effects of chemotherapy in participants with blood cancers	NCT03589729	II	Recruiting
	JQ1	Inducer	BRD4 inhibitor	A549 cell xenografts	^133^	N/A	N/A	N/A	N/A
Reductive -oxidative	APAP	Inducer	Blocking the system Xc–	A549 cells xenografts	^185^	Delirium in old age; delirium; coronary artery disease	NCT04093219	III	Recruiting
						Nephrectomy	NCT03365622	IV	Recruiting
						Acute respiratory distress syndrome	NCT04291508	II	Recruiting
						Ductus arteriosus in preterm infants	NCT04459117	II, III	Recruiting
						Fear	NCT05396677	N/A	Recruiting
	AOA	Inhibitor	Pan-transaminases inhibitor	Colitis induced in mice with DSS; chronic alcoholism in rats	^375,376^	N/A	N/A	N/A	N/A
	AUF	Inducer	TXNRD inhibitor; inhibiting GSH biosynthesis	Hemochromatosis model (*Hfe*^*−/−*^ mice)	^22^	Glioblastoma	NCT02770378	I, II	Completed
						Recurrent non-small cell lung cancer or small cell lung cancer	NCT01737502	I, II	Recruiting
						Chronic lymphocytic leukemia (CLL)	NCT01419691	II	Completed
						Recurrent epithelial ovarian; primary peritoneal, or fallopian tube cancer	NCT01747798	Early phase 1	Completed
	Brequinar	Inducer	DHODH inhibitor	HT-1080 xenografts; NCI-H226 xenografts and lung cancer PDXs	^20^	SARS-CoV-2 infection	NCT04575038	II	Completed
						Acute myeloid leukemia	NCT03760666	I, II	Terminated
	BSO	Inducer	Inhibition of GCL; GSH-depleting	MDA-MB-231 xenografts	^167^	Neuroblastoma	NCT00005835, NCT00002730	I	Completed
	CH004	Inducer	CBS inhibitor	H22 xenografts	^227^	N/A	N/A	N/A	N/A
	CUR	Inhibitor	GPX4 agonist	DSS-induced UC mice	^220^	N/A	N/A	N/A	N/A
	DHA	Inhibitor	Inactivating the PRIM2/SLC7A11 axis; suppressing GPX4, ferritinophagy	NCI-H23 xenografts	^285^	Polycystic ovary syndrome	NCT05465135	IV	Active, not recruiting
	Edaravone	Inhibitor	RTA	CSDS depression model in mouse; particulate Matter-induced lung inflammation model in mouse	^377,378^	Optic neuritis	NCT05540262	N/A	Recruiting
						Acute ischemic stroke	NCT02430350	III	Completed
						Nasopharyngeal carcinoma, brain necrosis	NCT01865201	II	Completed
						Myocardial infarction	NCT00265239	IV	Completed
						Cerebral infarction	NCT00200356	IV	Completed
						ALS	NCT00415519	III	Completed
	Erastin	Inducer	SLC7A11 inhibitor	A375 melanoma xenografts; Hepa1–6 cells xenografts	^34,379^	N/A	N/A	N/A	N/A
	Erastin-APAP	Inducer	Nrf2/HO-1 inhibitor	A549 xenografts	^185^	N/A	N/A	N/A	N/A
	Ferrostatin-1	Inhibitor	RTA	Auranofin-treated *Hfe*^*-/-*^ mice; DOX- and I/R-induced cardiomyopathy in mice; hepatic I/R mice; cisplatin-induced AKI mice	^3,22,82,268^	N/A	N/A	N/A	N/A
	IKE	Inducer	SLC7A11 inhibitor	SUDHL6 xenografts	^153^	N/A	N/A	N/A	N/A
	Liproxsatain-1	Inhibitor	RTA	MCD-induced NASH in mice; hepatic I/R-induced mice; acute renal failure mice	^5,109^	N/A	N/A	N/A	N/A
	NAC	Inhibitor	GSH synthesis regulator	Polycystic ovary syndrome model in rats; hemorrhagic stroke in mice; diabetic nephropathy model in beagle; intermittent hypoxia-induced myocardial injury in mic	^73,380–382^	Drug-resistant epilepsy	NCT05485558	II	Recruiting
						Autism spectrum disorder	NCT04278898	II	Recruiting
						Alcohol use disorder	NCT03707951	II	Recruiting
						Cannabis use disorder	NCT03055377	II, III	Recruiting
						Progressive MS	NCT05122559	II	Recruiting
						Neurofibromatosis 1	NCT04481048	II	Recruiting
						Cocaine addiction	NCT03423667	II	Recruiting
						Cannabis use disorder; tobacco use disorder; drug use disorder	NCT04627922	IV	Recruiting
						Skin disorder	NCT05287724	Early phase 1	Recruiting
						Chronic thromboembolic pulmonary hypertension	NCT04081012	N/A	Recruiting
						Mitochondrial disease	NCT05241262	I	Recruiting
						Diabetic neuropathies	NCT04766450	IV	Recruiting
						Bipolar disorder	NCT05340504	II	Recruiting
						Vascular cognitive impairment no dementia	NCT03306979	II	Recruiting
						Systemic lupus erythematosus (SLE)	NCT00775476	II	Recruiting
						Infertile women with endometrioma	NCT05460858	III	Recruiting
						Gaucher disease type 1	NCT02583672	II	Recruiting
						Alcohol use disorder; bipolar disorder	NCT03220776	II	Recruiting
						Mild cognitive impairment	NCT03493178	Early phase 1	Recruiting
	PE	Inducer	SLC7A11 inhibitor	HT-1080 cell xenografts	^13^	N/A	N/A	N/A	N/A
	RSL3	Inducer	GPX4 inhibitor	HT-1080 cell xenografts; MCD-induced NASH in mice; HepG2 cell xenografts	^13,109,205^	N/A	N/A	N/A	N/A
	SAS	Inducer	SLC7A11 inhibitor	Prostate cancer cell DU-145 and PC-3 xenografts; B16F10 melanoma xenografts; glioblastomas xenografts	^161,166,168^	Glioma; glioblastoma; recurrent glioblastoma	NCT04205357	I	Recruiting
						Breast cancer; chronic pain due to malignancy	NCT03847311	II	Recruiting
	TRG	Inducer	NRF2 inhibitor	HN3R xenografts; Hepa1–6 xenografts	^34,204^	N/A	N/A	N/A	N/A
	TRG+erastin +sorafenib	Inducer	Inhibiting NRF2 and system Xc^-^	Hepa1–6 xenografts	^34^	N/A	N/A	N/A	N/A
	UAMC-3203	Inhibitor	RTA	Ferroptosis model using acute iron poisoning	^264^	N/A	N/A	N/A	N/A
	WA	Inducer	Alkylation of GPX4	IMR-32 xenografts	^202^	Recurrent ovarian cancer	NCT05610735	I and II	Not yet recruiting
Lipid metabolism	Baicalein	Inhibitor	ALOX12 inhibitor; ALOX12/ALOX15 inhibitor; ACSL4 inhibitor	Heart I/R injury in rat; transient MCAO mice; myocardial I/R rat	^295,296,383^	Influenza	NCT03830684	II	Unknown
	IMA-1	Inhibitor	Perturbation of ALOX12-ACC1 interaction	HFHC-treated NASH in mice; spontaneous NASH in Cynomolgus macaques;	^258^	N/A	N/A	N/A	N/A
	ML355	Inhibitor	ALOX12 inhibitor	HFHC-treated NASH in mice; spontaneous NASH in Cynomolgus macaques	^258^	N/A	N/A	N/A	N/A
	NDGA	Inhibitor	Pan-LOX inhibitor	HFD-induced fatty liver in obese mice	^384^	Prostate cancer	NCT00678015	II	Terminated
	Pioglitazone	Inducer	ACSL4 inhibitor	BxPC-3 xenografts; HT-29 and SW480 xenografts	^385,386^	Breast cancer; muscle fatigue	NCT05013255	II	Recruiting
						Gastroparesis	NCT04300127	Early phase 1	Recruiting
						Chronic kidney diseases	NCT03471117	IV	Recruiting
						Uric acid nephrolithiasis	NCT04370093	IV	Recruiting
						NASH	NCT05254626	IV	Recruiting
						Cocaine use disorder	NCT04843046	II	Recruiting
						Alcohol use disorder	NCT05107765	I, II	Recruiting
	Rosiglitazone	Inducer	ACSL4 inhibitor	I/R-induced intestinal injury mice; renal *Gpx4*^*-/-*^ mice	^4,15^	Solid tumor malignancies	NCT04114136	II	Recruiting
						Prostate cancer	NCT00182052	III	completed
						Ulcerative colitis; inflammatory bowel disease	NCT00065065	II	completed
						HIV infection	NCT00367744	II	completed
						Sarcoma	NCT00004180	II	completed
						Alzheimer’s disease	NCT00688207	I	completed
						NASH	NCT00492700	II	completed
						Kidney transplant	NCT00309309	II	Completed
	PRGL493	Inhibitor	ACSL4 inhibitor	HCG treated mice; MDA-MB-231 and PC-3 xenografts	^387^	N/A	N/A	N/A	N/A
	Troglitazone	Inducer	ACSL4 inhibitor	MIA Paca2 cells	^388^	Sarcoma	NCT00003058	II	Completed
	Zileuton	Inhibitor	ALOX5 selective inhibitor	NaIO_3_-induced acute retinal degeneration in mice	^389^	Chronic myelogenous leukemia	NCT02047149	I	Terminated
						Chronic myelogenous leukemia	NCT01130688	I	Terminated
						Sickle cell disease	NCT01136941	I	Completed
						Head and neck cancer; lung cancer	NCT00056004; NCT00070486	II	Completed
						Tobacco use disorder	NCT02348203	II	Completed
						Tobacco use disorder	NCT01021215	I, II	Completed
						Acne vulgaris	NCT00098358	II	Unknown

Abbreviations: *ACC1* acetyl-CoA carboxylase 1, *ACSL4* acyl-CoA synthetase long-chain family member 4, *AKI* acute kidney injury, *ALOX* arachidonate lipoxygenase, *ALS* amyotrophic lateral sclerosis, *AOA* aminooxyacetic acid, *APAP* acetaminophen, *AUF* auranofin, *BRD4* bromodomain-containing protein 4, *BSO* buthionine sulphoximine, *CBS* cystathionine beta-synthase, *CPX* ciclopirox, *CSDS* chronic social defeat stress, *CUR* curculigoside, *DFO* deferoxamine, *DFP* deferiprone, *DFX* deferasirox, *DHA* dihydroartemisinin, *DHODH* dihydroorotate dehydrogenase, *DOX* doxorubicin, *DSS* dextran sulfate sodium, *DXZ* dexrazoxane, *Fth1* ferritin heavy chain 1, *GCL* glutamate-cysteine ligase, *GPNA L-g-*glutamyl-p-nitroanilide, *GSH* glutathione, *HFD* high-fat diet, *HFHC* high-fat/high-cholesterol, *Ho-1* heme oxygenase 1, *ICH* intracranial hemorrhage, *IKE* imidazole ketone erastin, *I/R* ischemia/reperfusion, *LPS* lipopolysaccharide, *MCAO* middle cerebral artery occlusion, *MCD* methionine/choline-deficient diet, *MS* multiple sclerosis, *N/A* not applicable, *NAC* *N*-acetylcysteine, *NASH* non-alcoholic steatohepatitis, *NCOA4* nuclear receptor coactivator 4, *NCT* national clinical trial, *NDGA* nordihydroguaiaretic acid, *NOD* nonobese diabetic, *Nrf2* nuclear factor erythroid 2-related factor 2, *PDTC* pyrrolidine dithiocarbamate, *PDXs* patient-derived xenografts, *PE* piperazine erastin, *RSL3* RAS-selective lethal small molecule 3, *RTA* radical trap antioxidant, *SAS* sulfasalazine, *SCID* severe combined immunodeficiency, *SLC* solute carrier family, *UC* ulcerative colitis, *TRG* trigonelline, *TXNRD* thioredoxin reductase, *WA* withaferin A

### Targeting dysregulated iron metabolism

**Fig. 3:**
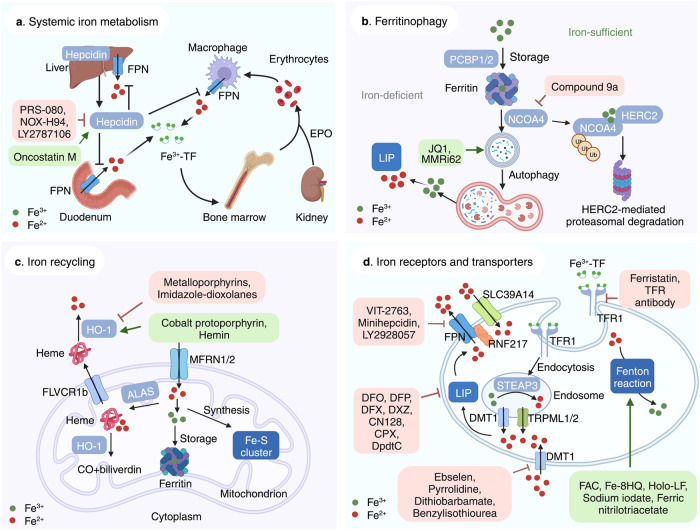
Targeting ferroptosis in iron metabolism. **a** Overview of systemic iron homeostasis. Labile iron binds to transferrin (TF) in the blood, and senescent erythrocytes are phagocytized by macrophages, releasing iron ions back into the circulation. The primary regulatory mechanism of iron homeostasis involves liver-derived hepcidin, which controls the cellular export of iron via ferroportin (FPN). **b** Overview of the various processes that involve ferritin under iron-deficient and iron-sufficient conditions. When cellular iron is sufficient, ferritin stores iron. Under iron-deficient conditions, ferritin undergoes NCOA4-mediated ferritinophagy and releases iron. **c** The active labile iron pool can be used either directly for incorporation into iron-containing proteins or transported into the mitochondria. **d** Overview of iron transporters in the plasma membrane and in lysosomes. Molecules in the pink and green text boxes are inhibitors or activators, respectively, of the pathways that regulate iron metabolism and suppress or trigger, respectively, ferroptosis. ALAS 5-amibolevulinic acid synthase, DMT1 proton-coupled divalent metal ion transporter 1, EPO erythropoietin; FLVCR1b, FLVCR heme transporter 1b, HERC2 HECT and RLD domain containing E3 ubiquitin protein ligase 2, HO-1 heme oxygenase 1, LIP labile iron pool, MFRN mitoferrin, NCOA4 nuclear receptor coactivator 4, PCBP poly(rC)-binding protein, RNF217 E3 ubiquitin protein ligase RNF217, SLC solute carrier family, STEAP 6-transmembrane epithelial antigen of the prostate metalloreductase family, TFR1 transferrin receptor protein 1, TRPML lysosomal cation channel mucolipin. Created with BioRender.com

Iron is essential to many physiological processes, including electron delivery, oxygen transport, and DNA biosynthesis. Iron is stored and transported as Fe^3+^, while Fe^2+^ serves as an electron donor to catalyze the Fenton reaction. During iron overload conditions, excess iron is involved in the generation of free radicals, lipid peroxidation, and DNA damage, which in turn promotes ferroptosis. At the cellular level, transferrin (TF) mediates the cellular uptake of Fe^3+^ via endocytosis of iron-loaded TF-bound transferrin receptor 1 (TFR1), followed by release from the endosome,^93,94^ in which Fe^3+^ is then converted to Fe^2+^ via the metalloreductase STEAP3.^95^ When the binding capacity of TF-2Fe^3+^ is saturated, ferrireductases reduce Fe^3+^ to Fe^2+^, which can be transported to the LIP via NTBI transporters such as DMT1 (divalent metal transporter 1)^96^ and SLC39A14.^97^ The LIP can also increase due to the degradation of either heme or hemoglobin, which are captured by hemopexin and haptoglobin, respectively. Upon binding to CD163 (a monocyte/macrophage-specific scavenger receptor)^98^ and LRP1 (LDL receptor related protein 1),^99^ heme-hemopexin and hemoglobin-haptoglobin complexes are internalized via endosomes, after which the rate-limiting enzyme heme oxygenase-1 (HO-1) catabolizes heme to produce biliverdin, releasing iron into the LIP. Iron is reserved primarily in ferritin, a complex consisting of 24 light chain (FTL) and heavy chain (FTH1) subunits.^100^ FTH also has ferroxidase activity, converting Fe^2+^ to Fe^3+^ to prevent iron-induced toxicity. Excess iron is exported by FPN, the body’s sole iron exporter,^101^ which is regulated primarily by the hepatic peptide hepcidin,^102^ the master regulator of iron homeostasis. Thus, together with our recent finding that the newly identified E3 ubiquitin ligase RNF217 mediates the degradation of FPN,^103^ these results indicate that systemic iron homeostasis is tightly controlled by the hepcidin-FPN axis, and targeting dysregulated iron metabolism may directly affect ferroptosis (Fig. 3).

#### Using iron chelators to inhibit ferroptosis

The term “ferroptosis” originated from the rescue effect of the iron chelator DFO. In the clinic, DFO, deferiprone (DFP), and deferasirox (DFX) are commonly used iron chelators.^104^ DFO is derived from *Streptomyces pilus* and is approved for managing iron overload‒related diseases such as hemochromatosis, thalassemia, and sickle cell anemia, as well as other chronic iron overload‒induced conditions such as blood transfusion.^105–107^ Although DFO has been reported to prevent ferroptosis in several animal disease models, including neurodegeneration, I/R-induced injury, NASH, and hemorrhagic stroke,^3,46,82,108,109^ DFO can cause toxic side effects such as anemia and edema.^110^ DFP, an effective, orally administered hydrophilic iron chelator, was developed as an alternative to DFO.^111^ Compared to DFO, DFP is relatively inexpensive and more effective, with lower toxicity, making DFP more suitable for use in iron-overloaded transfusion patients who currently cannot receive any form of chelation therapy.^112^ In addition, DFP can cross the blood-brain barrier (BBB) and can transfer iron in DFP-Fe to transferrin.^113,114^ DFP has been shown to improve motor function by decreasing iron content in the brain in patients with Friedreich’s ataxia.^115^

Similar to DFP, DFX is a highly selective iron chelator showing a high affinity for Fe^3+^ and a relatively long biological half-life.^116^ Studies have shown that DFX treatment can prevent the accumulation of hemosiderin (a complex composed of partially digested ferritin and lysosomes for iron storage) and can ameliorate ferroptosis-related kidney and neuronal damage.^117,118^

Currently, dexrazoxane (DXZ) is the only iron chelator approved by the FDA for protecting against doxorubicin (DOX)-induced cardiotoxicity by chelating DOX-induced mitochondrial iron.^119^ Compared to DXZ, the novel orally administered iron chelator CN128 is more potent and has fewer side effects and is currently being studied in a phase II clinical trial for treating β-thalassemia following regular blood transfusion.^120^ Finally, ciclopirox olamine, which is currently approved for treating cutaneous fungal infections, has also been used as an iron chelator in various model systems due to its inhibitory activity on iron-dependent ribonucleotide reductase.^121,122^

#### Iron receptors and transporters

The TF receptor TFR1 is a glycoprotein that interacts with iron-bound TF in order to mediate cellular iron uptake. Gao et al. reported that TFR1-mediated cellular uptake of TF-bound iron is required for ferroptosis, showing that inhibiting TFR1 using RNA interference (RNAi) can efficiently block ferroptosis.^25^ Moreover, Wu et al. showed that knocking down *TFR1* inhibits ferroptosis under cystine starvation conditions, whereas upregulating TFR1 significantly activates ferroptosis via the NF2-YAP signaling pathway.^123^ Using the TFR1-specific antibody 3F3-FMA, Feng and colleagues showed that TFR1 can serve as a marker for cells undergoing ferroptosis.^124^ Given that various cancer cells express relatively high levels of TFR1, antibodies that either neutralize of block TFR1 have been investigated as potential cancer therapies. For example, Horonchik and Wessling-Resnick found that the small-molecule TFR1 inhibitor ferristatin (also known as NSC306711) can inhibit iron uptake by mediating the degradation of TFR1(ref. ^125^). Based on these results, it is reasonable to speculate that TFR1 antibodies may be suitable for treating ferroptosis-induced diseases, warranting further study.

DMT1 (also known as SLC11A2, DCT1, and NRAMP2) is known for its ability to transport Fe^2+^ into the duodenum and out of the endosome during the TF cycle, but it can also transport NTBI.^96^ DMT1 inhibitors such as ebselen,^126^ pyrrolidine dithiocarbamate (PDTC),^126^ and benzylisothiourea^127^ have been shown to reduce iron-induced damage by potently reducing the DMT1-mediated cellular uptake of NTBI, indicating that DMT1 may be a promising target for regulating ferroptosis to manage ferroptosis-related diseases.

Two strategies have been suggested to modulate ferroptosis by targeting iron export, namely hepcidin agonists such as minihepcidins^128^ and FPN inhibitors such as VIT-2763 (ref. ^128^). Similarly, small molecules that directly upregulate hepcidin expression may affect ferroptosis by promoting intracellular iron accumulation. In addition, endogenous inducers of hepcidin such as the cytokine oncostatin M (encoded by the *OSM* gene)^129^ may promote ferroptosis. Conversely, clinical trials involving hepcidin antagonists such as PRS-080,^128^ NOX-H94,^130^ and LY2787106^131^ may suppress ferroptosis by reducing intracellular iron content. Thus, further studies are needed in order to ascertain the functional role of these FPN/hepcidin regulators in modulating ferroptosis under pathological conditions.

#### Ferritinophagy

Under iron-deficient conditions, NCOA4 binds to iron-loaded ferritin, thus promoting lysosomal ferritin degradation and releasing iron into the LIP, a process known as ferritinophagy.^132^ Ferritinophagy has been shown to initiate ferroptosis via iron overload and lipid peroxidation.^26,27^ JQ1, a thienotriazolodiazepine that inhibits bromodomain proteins such as BRD4, has been reported to potentiate ferroptosis via ferritinophagy in breast cancer cells.^133^ Additionally, DpdtC (2,2′-di-pyridylketone dithiocarbamate) mobilizes iron by inducing ferritinophagy and can increase ferritinophagy-induced ROS production in MGC-803 cells, a human gastric carcinoma cell line.^134^ MMRi62, a small molecule compound initially identified as an inducer of apoptosis, is shown to potently trigger ferroptotic cell death in pancreatic ductal adenocarcinoma cells by increasing lysosomal ferritinophagy.^135^ On the other hand, Fang et al. recently found that a novel compound called 9a potently suppresses ferritinophagy-induced ferroptosis by competitively binding to NCOA4 and perturbing the interaction between NCOA4 and FTH1.^136^

#### Iron recycling by macrophages

Macrophages in the spleen and Kupffer cells (also known as stellate macrophages) in the liver maintain iron homeostasis by recycling iron obtained from senescent erythrocytes and damaged cells.^137^ Macrophage-mediated iron recycling is processed primarily in the spleen, and recycling iron is returned to the storage pool for utilization in various processes such as heme biosynthesis and Fe/S cluster formations in the mitochondria. The enzyme HO-1, encoded by the *HMOX1* gene, catalyzes heme to produce carbon monoxide, biliverdin, and free iron; biliverdin and free iron can be used to generate bilirubin and sequestered by ferritin, respectively. Upregulation of *HMOX1* expression has been shown to play a cytoprotective role^138^ and to increase the resistance of HCC cells to ferroptosis mediated by the p62-KEAP1 (Kelch-like ECH-associated protein 1)-NRF2 (nuclear factor erythroid-2-related factor 2) pathway.^34^ On the other hand, high *HMOX1* expression can also be toxic due to high levels of ferrous iron,^139^ which in turn accelerates the Fenton reaction, particularly in the context of insufficient levels of free radical scavengers. Together, these reports support the notion that *HMOX1* expression has a dose-dependent differential role.^140^ Targeting HO-1 has been proposed as a viable strategy for treating many diseases and conditions, including cardiovascular disease^3^ and inflammation.^141^ Notably, we previously showed that inhibiting HO-1 prevents ferroptosis-induced cardiomyopathy in mice.^3^ To date, many HO-1 agonists and antagonists have been proposed for use in various disease models. For example, Vreman et al. found that metalloporphyrins—synthetic heme analogs used to treat jaundice in newborn infants—can inhibit HO-1.^142^ However, several metalloporphyrins can induce phototoxicity and/or off-target adverse effects; given this poor safety profile, azalanstat was subsequently developed as a safer HO-1 inhibitor;^143^ however, studies are needed to test its efficacy in ferroptosis-related diseases.

### Targeting reductive-oxidative pathways

**Fig. 4:**
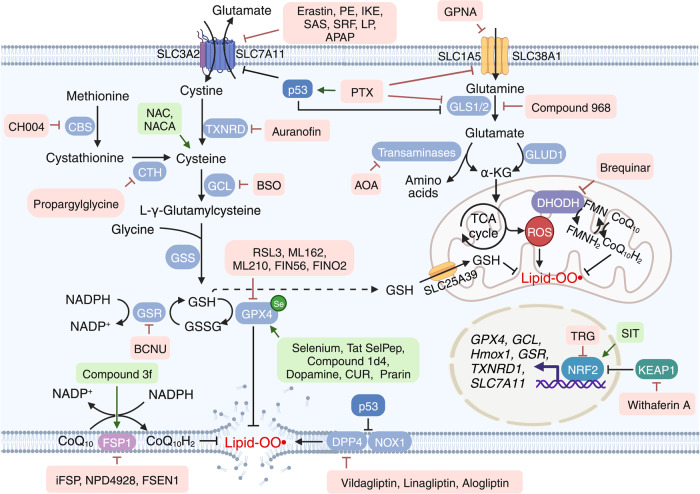
Targeting ferroptosis in reductive-oxidative pathways. Ferroptosis is tightly associated with levels of reactive oxygen species (ROS); therefore, homeostasis of the cellular reductive-oxidative response is important for regulating ferroptosis. The system Xc^–^-GSH-GPX4 pathway is a major ROS scavenger, and numerous molecules are designed to target the components involved in this pathway in order to modulate ferroptosis. The recently identified FSP1-CoQ_10_-NAD(P)H pathway and mitochondrial DHODH-mediated pathway are also potential targets for modulating ferroptosis. Moreover, NRF2 respond to cellular oxidative status by activating the transcription of genes involved in reductive-oxidative responses. Thus, targeting the KEAP1-NRF2 axis may be a viable strategy for modulating ferroptosis. Molecules listed in pink and green text boxes inhibit or induce, respectively, the indicated reductive-oxidative regulatory pathways, thereby suppressing or triggering, respectively, ferroptosis. CBS cystathionine beta-synthase, CoQ_10_ coenzyme Q_10_, CTH cystathionine gamma-lyase, DHODH dihydroorotate dehydrogenase, DPP4 dipeptidyl peptidase 4, FMN flavin mononucleotide, FMNH_2_ reduced flavin mononucleotide, FSP1 ferroptosis suppressor protein 1, GCL glutamate-cysteine ligase, GLS glutaminase, GLUD1 glutamate dehydrogenase 1, GPX4 glutathione peroxidase 4, GSH glutathione, GSR glutathione disulfide reductase, GSS glutathione synthetase, GSSG glutathione disulfide, KEAP1 Kelch-like ECH-associated protein 1, NOX1 NADPH oxidase 1, NRF2 nuclear factor erythroid 2-related factor 2, SLC solute carrier family, TCA tricarboxylic acid, TXNRD thioredoxin reductase. Created with BioRender.com

Under physiological conditions, ROS production is controlled by a coordinated network of antioxidative pathways. Among the various cellular antioxidative defense systems, the system Xc^–^-GSH-GPX4 metabolic pathway plays a key role in regulating ferroptosis. Intracellular cysteine is taken up primarily in the form of cystine via system Xc^–^^144^ or converted from methionine via the transsulfuration pathway,^145^ or is transported directly by alanine/serine/cysteine transporters (known as system ASC).^146,147^ In addition, the KEAP1/NRF2 antioxidative signaling pathway, the glutaminolysis pathway, the FSP1-CoQ_10_-NAD(P)H pathway, and the recently identified DHODH-mediated pathway have all been recognized as central mediators of ferroptosis by directly regulating reductive-oxidative pathways. Below, we summarize these ferroptosis modulators that target the reductive-oxidative pathways (see Fig. 4).

#### Pharmacological perturbation of the system Xc^–^-GSH-GPX4 pathway

Extracellular cystine imported by system Xc^–^ is subsequently reduced to cysteine, the rate-limiting precursor of GSH,^148,149^ an essential cofactor for the antioxidant enzyme GPX4, which quenches PL hydroperoxides. GSH is produced from cysteine, glutamate, and glycine exclusively in the cytosol via glutamate-cysteine ligase and glutathione synthetase. Newly synthesized cytosolic GSH is then actively transported into the mitochondria via the recently identified GSH importer SLC25A39.^150^ Moreover, studies have shown that inhibiting either system Xc^–^ or GPX4 potently induces ferroptosis via cysteine deprivation,^1^ driving GSH depletion and ultimately impairing the cell’s antioxidant defense mechanism.^151^

##### Triggering ferroptosis using system Xc^–^ inhibitors

Erastin is a first-generation ferroptosis inducer that directly suppresses system Xc^–1^. Although the pro-ferroptotic effects of erastin have been reported in many types of malignant cancer cells in vitro, its further development has been limited due to its low efficacy in vivo. To optimize the chemical properties of erastin, Yang et al. developed a more effective erastin derivative, piperazine erastin, by inserting a piperazine group in the aniline ring of erastin, yielding improved drug-like properties such as increased water solubility and metabolic stability.^13^ Piperazine erastin was subsequently shown to reduce tumor growth in an HT-1080 xenograft mouse model, with no apparent toxicity.^13^ However, piperazine erastin showed limited activity against established tumors, perhaps due to its moderate potency.^152^ To overcome this limitation, imidazole ketone erastin (IKE) was developed and shown to have high (nanomolar) potency and high metabolic stability.^152^ The tumor-suppressing efficacy of IKE was then demonstrated in SUDHL6 (diffuse large B cell lymphoma) cell xenografts in mice.^153^ Capitalizing on the fact that IKE is soluble under acidic aqueous conditions, nanoparticles were used as an IKE delivery system to further improve its therapeutic index, with reduced toxicity compared to free IKE.^153^ Previous studies found that erastin was able to increase the sensitivity of various cancer cell lines to chemotherapy drugs, including doxorubicin, actinomycin D, cisplatin, temozolomide, and cytarabine,^154–157^ providing new motivation for exploring the feasibility of combination therapies. Recently, we solved the high-resolution structure of erastin-bound human system Xc^–^ and showed that IKE is a highly potent inducer of ferroptosis, providing a structural basis for designing more effective ferroptosis modulators.^158^

The anti-rheumatic drug sulfasalazine (SAS) is clinically approved for treating inflammatory arthritis and inflammatory bowel disease. SAS has been reported to suppress the growth of lymphoma cells both in vitro and in vivo through the activation of ferroptosis by suppressing system Xc^–^(ref. ^159^). Moreover, a lot of studies have shown that SAS has anti-tumor activity in various xenograft tumor models, including glioblastoma,^160^ prostate cancer,^161^ small cell lung cancer,^162^ pancreatic cancer,^163^ and TNBC.^164^ Based on its excellent safety profile in animal studies, several phase I and phase II clinical studies have been initiated. However, various doses of SAS failed to produce a clinical response in malignant glioma, and side effects were reported, including anorexia, gastrointestinal toxicity, and hematological toxicity.^165^ In addition, long-term high-dose (8–12 g/day) treatment with SAS can induce several adverse side effects and should be avoided; therefore, combination therapy using SAS together with conventional radiotherapy and/or chemotherapy has been proposed as a promising therapeutic strategy.^166–173^ To mitigate its toxicity, studies involving SAS focus primarily on sensitizing agents and applications using nanoparticles, a novel strategy designed to increase the effectiveness of low-dose SAS.^174^

Sorafenib (SRF), a small molecular kinase inhibitor, is approved for treating various solid tumors, has well-characterized clinical efficacy and tolerability, and remains the only drug approved to treat advanced HCC. SRF has been shown to trigger ferroptosis in cancer cells by suppressing system Xc^–^.^175,176^ However, the relatively limited clinical benefits of SRF and the emergence of drug resistance are major hurdles hampering its further development. On the other hand, several studies found that combination therapies can increase SRF’s anti-tumor activity,^177,178^ as well as GSH starvation‒based nanoscience for cancer therapy.^179,180^ Notably, however, Zheng et al. recently reported that SRF failed to increase ferroptosis in various cancer cell lines.^181^ Thus, the precise role of SRF in ferroptosis, and its clinical value in the context of cancer, remains controversial and poorly understood.

Interestingly, a subset of FDA-approved drugs such as the muscle relaxant lanperisone and the analgesic acetaminophen (N-acetyl-p-aminophenol, or APAP) have been shown to activate ferroptosis by functionally suppressing system Xc^–^. For example, lanperisone has been shown to effectively target *RAS*-mutated cancers in vivo without causing overt toxicity.^182^ APAP is widely used as an analgesic and antipyretic, but can cause dose-dependent hepatotoxicity. Interestingly, Yamada et al. recently showed that APAP-induced hepatotoxicity is attributed to GSH depletion‒induced ferroptosis and proposed this as the predominant mechanism,^183^ providing new insights into the potential use of APAP in triggering ferroptosis in order to inhibit tumor growth. Indeed, several studies have shown a synergistic therapeutic effect of combining erastin and APAP to treat melanoma and lung cancer xenografts.^184,185^

##### GSH synthesis regulators

GSH has a central role in the protection of cells from oxidative damage and toxic reactive species. Therefore, targeting pathways involved in GSH synthesis has been studied extensively as a major strategy for treating ferroptosis-related diseases.

Auranofin (AUR) is a gold (I)‒containing compound used to treat rheumatic arthritis. Recent studies have shown that AUR has therapeutic potential for other diseases and conditions such as cancer, metabolic disease, and infectious and inflammatory diseases.^186^ Indeed, we provided the first report that AUR can be used in vivo as to induce ferroptosis in mice by suppressing the activity of thioredoxin reductase,^22^ providing compelling evidence supporting its further clinical testing—either alone or combined with other therapies—for treating ferroptosis-resistant disease conditions such as cancer. To expand the potential clinical applications of AUR, several studies have investigated repurposing AUR; for instance, AUR has been repurposed as an anticancer drug in TNBC cells by inhibiting GSH biosynthesis.^187^ Recently, we found that malic enzyme 1 (Me1) regulates ferroptosis in hepatic I/R-induced injury, and its inactivation further decreased the production of NADPH and failed to restore GSH synthesis,^188^ suggesting that targeting Me1 may be a viable strategy for treating ferroptosis-related diseases by altering GSH synthesis.

Buthionine sulfoximine (BSO) activates ferroptosis by inhibiting glutamate-cysteine ligase (GCL).^189^ Similar to erastin, BSO has also been used to deplete GSH and induce ferroptosis in several cancer cell lines.^13,41^ Based on early clinical trials, BSO is considered safe but has limited therapeutic benefit for treating refractory malignancies.^190^ To improve its clinical efficacy, researchers attempted to identify BSO-sensitive cancer types and patients, revealing that low GSH levels can serve as a marker for BSO susceptibility.^191^

1,3-bis-(2-chloroethyl)-1-nitrosourea (BCNU, also known as carmustine) is a selective glutathione disulfide reductase (GSR) inhibitor^192^ and is used as a chemotherapy drug for treating brain cancer and lymphoma. Based in its mode of action, BCNU is believed to induce ferroptosis by directly inhibiting GSH synthesis. Recently, a combination of BCNU and sorafenib was shown to significantly suppress the in vivo growth of liver cancer by promoting ferroptosis.^193^ In addition, both BCNU and AUR have been shown to cause cell death in oxidant-enriched tumorigenic endothelial (EOMA) cells,^194^ possibly by activating ferroptosis. These findings may therefore increase the clinical application of BCNU for the treatment of HCC and endothelial cell tumors.

*N*-acetylcysteine (NAC)^195^ is clinically approved to treat APAP overdose. As an antioxidant, NAC has been shown to inhibit ferroptosis by targeting cysteine metabolism. In addition to protecting the liver from APAP-induced ferroptosis, NAC has also been clinically shown to improve neurodegeneration-related symptoms by increasing cysteine levels and facilitating the synthesis of γ-glutamyl-cysteine and GSH.^196^ Due to its poor bioavailability, however, NAC must be administered in fairly high doses and requires a long treatment time in patients with severe APAP overdose, thereby increasing the risk of an anaphylactoid reaction (i.e., nonimmunologic anaphylaxis), fluid overload, and high fluid osmolarity.^197^ To overcome these issues, *N*-acetylcysteine amide (NACA), a modified form of NAC with increased membrane permeability^198^ and bioavailability (67% compared to only 15% for NAC),^199^ has been developed. To date, NACA has been shown to have antioxidant activity in several preclinical models, but is yet to be approved for clinical use.^200^

##### Promoting ferroptosis using GPX4 inhibitors

The essential antioxidant enzyme GPX4 is not only responsible for maintaining redox homeostasis, but is also recognized as the “ferroptosis gatekeeper” by transforming lipid ROS into lipid alcohols. Direct inactivation of GPX4 has been shown to drive ferroptosis independent of intracellular cysteine and GSH levels.^13^ Thus, numerous studies have focused on identifying novel activators of ferroptosis by targeting GPX4 in order to develop new cancer therapeutics.

As a covalent inhibitor of GPX4, RSL3 was originally identified as a pro-ferroptosis compound through chemical screening.^1^ Although sharing the more common features of ferroptosis, RSL3-mediated ferroptosis does not include decreased levels of cellular GSH. GPX4 was subsequently identified as the primary target of RSL3 using an unbiased, affinity-based chemoproteomics approach.^13^ Moreover, genetically knocking down and overexpressing *GPX4* were shown to cause sensitization and resistance to RSL3, respectively, providing further evidence that GPX4 is the target of RSL3.^13^ Since these previous studies, RSL3 has been widely used as a ferroptosis agonist in vitro in a wide range of cancer cell types, including pancreatic cancer, adrenocortical carcinoma, fibrosarcoma,^201^ and breast cancer^15^ cells. In addition, RSL3 may also serve as a chemosensitizer for cisplatin, doxorubicin, and actinomycin D by activating ferroptosis in various cancer cells, including neuroblastoma,^202^ osteosarcoma,^155^ lung cancer,^203^ and rhabdomyosarcoma cell lines.^154^ In mouse xenograft models, RSL3 is well-tolerated, with no overt toxicity or body weight loss observed even at the relatively high dose of 400 mg/kg.^13,204,205^ As an experimental tool, RSL3 has been used extensively to study the role of ferroptosis in various disease models, including AKI, I/R-induced injury, and neurodegenerative disease.^206–208^

Similar to RSL3, ML162 was identified as a GPX4 inhibitor,^209^ and subsequent studies showed that ML162 can directly bind GPX4 and inhibit its activity.^13^ However, both RSL3 and ML162 have relatively poor selectivity and pharmacokinetics, as they covalently bind to GPX4 via a reactive alkyl chloride moiety.^210^ To develop a new GPX4 inhibitor containing a different moiety, Eaton et al. used masked nitrile-oxide electrophiles to create ML210, a more selective covalent suppressor of GPX4 with improved pharmacokinetics and comparable activity.^211^ However, given that the in vivo efficacy of all GPX4 inhibitors developed to date remains limited, efforts have focused on developing more efficacious GPX4-selective inhibitors.

Based on the structural optimization of CIL56 (caspase-independent lethal 56), FIN56 was developed as a highly selective inducer of ferroptosis that promotes GPX4 degradation as well as the GPX4-independent activation of squalene synthase (SQS).^212^ FIN56 potently induces ferroptosis in all TNBC cell lines tested to date, suggesting that FIN56 may be an effective additional pro-ferroptosis anticancer drug.^213^ In contrast, the endoperoxide-containing 1,2-dioxolane FINO_2_ was shown to potently and selectively induce ferroptosis in engineered cancer cells by directly oxidizing iron and indirectly inactivating GPX4.^214^ In addition, the naturally occurring C28 steroidal lactone withaferin A (WA), one of the best-studied withanolides, has a diverse range of pharmacological activities, including anti-inflammatory, anti-tumor, and antioxidant properties. In both high-risk neuroblastoma cell lines and neuroblastoma xenografts, WA has been shown to dose-dependently induce ferroptosis via the dual mechanisms of inactivating GPX4 and activating the NRF2 pathway.^202^ Thus, natural products may serve as an additional resource for the discovery of new GPX4 inhibitors.

Recently, Wu et al. identified creatine kinase B (CKB)-enhanced GPX4 as a novel mechanism for regulating ferroptosis, accounting for the resistance of HCC cells to ferroptosis by stabilized GPX4 via CKB phosphorylated GPX4 at residue S104.^215^ Mutations in either CKB or GPX4 significantly increase the ferroptosis-mediated cell death of HCC by inhibiting this phosphorylation.^215^ Importantly, the authors showed a positive correlation between phosphorylated GPX4 and HCC aggressiveness in clinical samples,^215^ suggesting a potential new strategy for treating HCC by blocking CKB/GPX4-mediated ferroptosis.

Although GPX4 inhibitors potently inhibit cells growth in vitro,^216^ all currently identified GPX4 inhibitors have limited prospects for further clinical development due to their poor pharmacokinetics and specificity. Therefore, more studies are needed in order to develop GPX4-specific inhibitors with improved pharmacological properties.

##### Suppressing ferroptosis using GPX4 activators

The essential micronutrient selenium (Se) is required for the production of selenocysteine,^217^ which serves as the active site for GPX4.^16^ Ionic selenite (SeO_3_^2-^) is commonly used to deliver Se to cultured cells. For example, treating cultured neurons with SeO_3_^2-^ was shown to increase *GPX4* transcription and inhibit ferroptosis induced by either hemin or homocysteic acid.^74^ In addition, systemically treating mice with Tat SelPep, a selenocysteine-containing peptide that can cross the BBB, was shown to improve functional recovery following hemorrhagic and ischemic stroke by blocking ferroptosis.^74^ Together, these studies suggest that Se supplementation may help protect against ferroptosis-related tissue damage and disease.

The neurotransmitter dopamine has many physiological roles, particularly in controlling various functions in the central nervous system, including movement, memory, motivation, mood, and attention. Insufficient levels of dopamine production are known to contribute to the progression of PD, and dopamine-based therapies such as levodopa and dopamine receptor agonists have been used clinically to treat PD and various cardiovascular conditions. Interestingly, non-oxidative dopamine was reported to potently inhibit erastin-induced ferroptosis in both cancerous and non-cancerous cells by stabilizing GPX4,^218^ suggesting that dopamine may be a promising candidate drug for alleviating ferroptosis-related tissue injury and disease, as well as certain neurodegenerative diseases.

Using a combination of computational prediction and experimental validation, Li et al. discovered eight new potential GPX4 activators and found that compound 1d4 was the most potent allosteric activator of GPX4, significantly increasing GPX4 activity by 50% when applied at 20 μM in a cell-free assay and 61 μM in cell extracts; moreover, they found that compound 1d4 potently inhibited ferroptosis when applied to HT-1080 fibrosarcoma cells.^219^

The diterpenoid triepoxide curculigoside is one of the primary bioactive phenolic compounds isolated from *Curculigo orchioides Gaertn* (commonly known as “Kali Musli”), an ancient medicinal plant well known for its immunomodulatory and rejuvenating effects. Recently, Wang et al. reported that curculigoside inhibits ferroptosis in IEC-6 cells (a cell line used to model intestinal iron transport) by upregulating *GPX4* expression.^220^ In mice with dextran sulfate sodium (DSS)‒induced ulcerative colitis, curculigoside was shown to protect against disease progression by reducing ferroptosis via GPX4 induction,^220^ suggesting that activating GPX4 is a viable therapeutic option for ulcerative colitis. Another plant-derived bioactive compound, puerarin, is an isoflavone glycoside isolated from *Pueraria lobata* (also known by its Chinese name, Gegen) with various pharmacological effects,^221^ including antioxidant, anticancer, and anti-inflammation properties, in addition to alleviating pain, promoting bone formation, attenuating insulin resistance, and exerting cardiac and neuronal protective effects. However, its molecular target and mechanism remain unknown. Interestingly, the anti-ferroptosis activity of puerarin in heart disease has been linked to the induction of FTH1 and GPX4 in rats.^222^ Given that the majority of bioactive natural compounds have relatively low bioavailability, future studies should focus on developing rationally designed combination therapies and/or their incorporation in nanoparticles.

#### Targeting the p62-KEAP1-NRF2 signaling pathway

The p62-KEAP1-NRF2 signaling pathway plays a regulatory role in response to oxidative stress, environmental insult, and toxic chemicals. In response to oxidative stress, p62 activates NRF2 by directly binding to its ubiquitin ligase adapter KEAP1; as a result, NRF2 translocates to the nucleus and regulates cellular redox homeostasis by modulating the expression of target genes, including several genes encoding enzymes involved in both GSH synthesis and iron homeostasis.^223^ Thus, NRF2 signaling is important for regulating ferroptosis.

The polar hydrophilic alkaloid trigonelline can be extracted from many plant species, including coffee beans and fenugreek seeds, and has been shown to significantly sensitize cancer cells to ferroptosis inducers by inhibiting NRF2.^34,204,224^ In addition, sitagliptin, a selective inhibitor of dipeptidyl peptidase 4 (DPP4) used to treat type 2 diabetes, was shown recently to inhibit ROS production, inflammation, and excessive autophagy by promoting Nrf2 translocation to the nucleus in mouse models of acute lung injury induced by severe pancreatitis.^225^ In addition, a modest dose of WA was shown to induce ferroptosis and increase the LIP by directly targeting KEAP1, mediating the NRF2-mediated upregulation of HO-1.^202^

#### Additional reductive-oxidative pathways involved in ferroptosis

Other reductive-oxidative pathways, including the transsulfuration, FSP1-CoQ_10_-NAD(P)H, glutaminolysis, and DHODH-mediated pathways, have also been linked to ferroptosis and are discussed in detail below.

##### The transsulfuration pathway

When cysteine availability is restricted, the transsulfuration pathway is activated in order to increase the synthesis of cysteine from methionine. The enzyme cystathionine beta-synthase (CBS) activates the first step in this pathway, followed by the formation of cystathionine into cysteine by the enzyme cystathionine gamma-lyase (CTH). Sustained activation of the reverse transsulfuration pathway has been implicated in the resistance of ovarian cancer cells to erastin-induced ferroptosis.^226^ CH004, a pharmacological inhibitor of CBS, has been identified as a novel stimulator of ferroptosis in liver cancer both in vitro and in vivo,^227^ providing experimental evidence to support the therapeutic potential of targeting the transsulfuration pathway in liver cancer. Similarly, the CTH inhibitor propargylglycine has been shown to sensitize NSC-34 cells to both erastin- and RSL3-induced ferroptosis.^228^ In mice, propargylglycine was also shown to increase susceptibility to APAP-induced liver damage and mortality,^229^ possibly by inducing ferroptosis.

##### The FSP1-CoQ_10_-NAD(P)H pathway

The *FSP1* gene was initially identified as a pro-apoptotic gene,^230^ but has also been identified as an anti-ferroptosis gene via FSP1’s oxidoreductase activity, which reduces CoQ_10_ to ubiquinol (CoQ_10_H_2_).^17,18^ Moreover, the FSP1 inhibitor iFSP1 was shown to selectively activate ferroptosis in *GPX4*-knockout *FSP1-*overexpressing cells and was identified by screening 10,000 drug-like compounds;^17^ iFSP1 was also shown to potently increase the sensitivity of cancer cells to RSL3-induced ferroptosis.^17^ Recently, Yoshioka et al. identified the novel compound called NPD4928 as an FSP1 inhibitor;^231^ the authors then showed that NPD4928 increased the cytotoxicity of various cell types in response to GPX4 antagonists, indicating that NPD4928 and GPX4 inhibitors may work synergistically to treat cancer by inducing ferroptosis.^231^ Most recently, Hendricks et al. developed another FSP1 inhibitor called FSEN1, which they found sensitized various cancer cells to ferroptosis.^232^ Together, these findings suggest that pharmacologically inhibiting the FSP1-CoQ_10_-NAD(P)H pathway may provide a plausible strategy for sensitizing cancer cells to ferroptosis-resistant therapeutic agents. On the other hand, Fang et al. recently identified the diphenylbutene derivative compound 3f as a ferroptosis inhibitor, and they showed that this compound can protect against ischemic stroke in rats by increasing FSP1 protein levels,^233^ suggesting that novel agents that upregulate FSP1 may be used to treat ferroptosis-related diseases.

##### The glutaminolysis pathway

Cellular uptake of the amino acid glutamine (Gln) is mediated primarily by specific transporters such as SLC38A1 and SLC1A5. Following its cellular uptake, intracellular Gln is converted by glutaminase (GLS) to produce glutamate, which can be further catalyzed to α-ketoglutarate either by glutamate dehydrogenase (GLUD1)‒mediated deamination or by transaminase-mediated transamination.^234^ Given that this reaction promotes ferroptosis by causing an accumulation of lipid peroxidation,^25^ blocking the glutaminolysis pathway has been proposed as a potential therapeutic approach for treating ferroptosis-induced tissue damage.

Inhibiting glutaminolysis using L-g-glutamyl-p-nitroanilide (GPNA, an inhibitor of the SLC38A1/SLC1A5 complex), compound 968 (an inhibitor of GLS), or amino-oxyacetic acid (AOA, an inhibitor of pan-transaminases) has been shown to block cystine deprivation‒induced ferroptosis in mouse embryonic fibroblasts, melanoma cells, as well as head and neck cancer cells.^25,235,236^ In addition, compound 968 was shown to potently prevent I/R-induced heart damage in an ex vivo model.^25^

Recent studies showed that low-concentration paclitaxel (PTX, a mitotic chemotherapeutic drug) suppressed cancer cell proliferation by promoting the production of lactate and changing the pH of the tumor microenvironment by downregulating glutaminolysis-related genes such as *GLS*, *SLC7A11*, and *SLC1A5*.^237,238^ In addition, low-dose PTX was shown to trigger ferroptosis by decreasing *SLC7A11* expression by upregulating the well-characterized transcription factor p53(ref. ^238^). Moreover, a growing body of evidence suggests that p53 regulates ferroptosis either by transcriptional or posttranslational mechanisms such as modulating the downstream expression of *SLC7A11*,^239^ *SAT1* (encoding spermidine/spermine N1-acetyltransferase 1),^240^ *GLS2* (encoding glutaminase 2),^25^ and *CDKN1A/p21* (encoding cyclin dependent kinase inhibitor 1A),^241^ or by directly inhibiting DPP4 activity in colorectal cancer cells.^242^

##### The DHODH-mediated pathway

The flavin-dependent mitochondrial enzyme DHODH catalyzes the de novo synthesis of pyrimidine.^243^ Originally, targeting DHODH was shown clinically to improve autoimmune diseases such as multiple sclerosis and rheumatoid arthritis. Although functional studies characterized DHODH as a potential target for treating cancer, several potent DHODH suppressors such as brequinar, leflunomide, and teriflunomide were clinically assessed but failed to receive FDA approval. Importantly, however, brequinar was recently shown to act synergistically with the FDA-approved drug SAS to suppress tumor growth by potently inducing ferroptosis,^20^ suggesting that DHODH may indeed serve as a viable target for treating cancer. Interestingly, DHODH inhibitors have also been developed as antiviral agents to act against cytomegalovirus, Ebola, influenza, and SARS-CoV-2(refs. ^244,245^). Whether ferroptosis is involved in the multifaceted activities of DHODH inhibitors remains an open question; nevertheless, targeting DHODH may have promise as a novel therapeutic approach for cancer and certain viral infections.

### Targeting lipid metabolic pathways

**Fig. 5:**
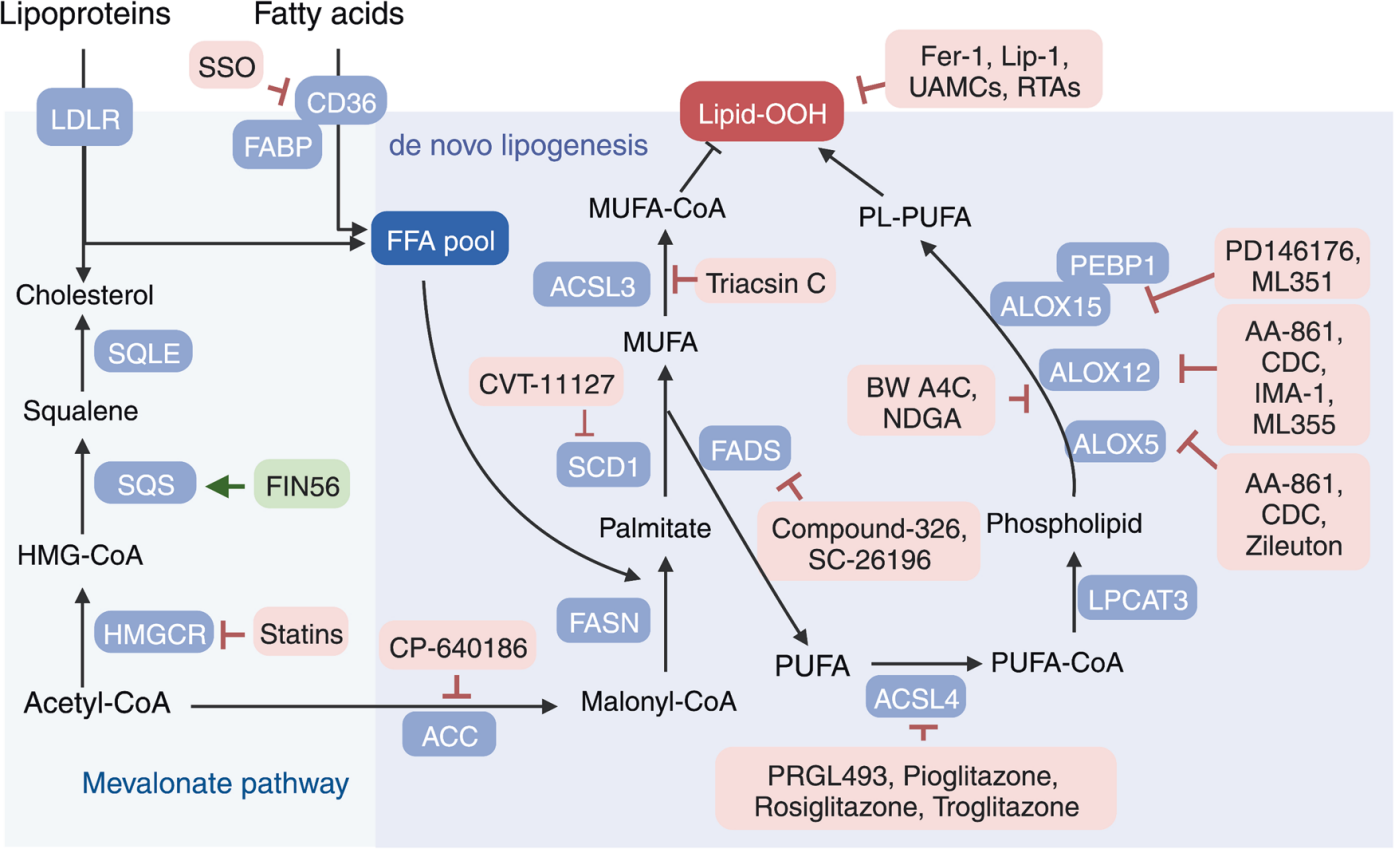
Targeting ferroptosis in lipid metabolism pathways. Cellular fatty acids are taken up by CD36 and FABPs, and stored in the free fatty acid (FFA) pool. Cells can also take up cholesterol via the LDLR or produce it from acetyl-CoA. These fatty acids can then be elongated to form long fatty acids and can be unsaturated to form monounsaturated fatty acids (MUFAs) or polyunsaturated fatty acids (PUFAs). MUFAs can be converted to PUFAs via the activity of FADS. ACSL4 and LPCAT3 are key enzymes that promote the incorporation of PUFAs into phospholipids (PLs) to form PL-PUFAs and induce ferroptosis. Molecules in the pink and green text boxes inhibit or induce, respectively, the indicated components in the lipid metabolism regulatory pathways, thereby suppressing or triggering, respectively, ferroptosis. ACC acetyl-CoA carboxylase, ACSL4 acyl-CoA synthetase long-chain family member 4, ALOX arachidonate lipoxygenase, CD36 cluster differentiation 36, FABP fatty acid binding protein, FADS fatty acid desaturase, FASN fatty acid synthase, HMG-CoA 3-hydroxy-3-methylglutaryl-CoA, HMGCR 3-hydroxy-3-methylglutaryl-CoA reductase, LDLR low density lipoprotein receptor, LPCAT3 lysophosphatidylcholine acyltransferase 3, PEBP1 phosphatidylethanolamine binding protein 1, SCD1 stearoyl-coenzyme A desaturase 1, SQLE squalene epoxidase, SQS squalene synthase. Created with BioRender.com

Lipid metabolism has an important regulatory role in the balance between cell survival and cell death. In ferroptosis, the majority of oxygenated PL species are upregulated, resulting in damage to PL-containing cell membranes. Four types of PLs—namely, the double and triple oxygenated arachidonic acid (AA)- and adrenic acid (AdA)-containing phosphatidylethanolamine (PE) species (C18:0/C20:4 and C18:0/C22:4)—were previously identified as the most susceptible substrates for lipid peroxidation.^30^ In addition to PE, other PLs can also be oxidized upon the induction of ferroptosis. Because lipid metabolic pathways are important for regulating lipid peroxidation, targeting these pathways may provide a novel strategy for treating ferroptosis-related diseases (Fig. 5).

A haploid genetic screen in KBM7 cells (a chronic myeloid leukemia cell line) revealed that the enzymes ACSL4 and LPCAT3 serve as ferroptosis regulators.^29^ ACSL4 and LPCAT3 catalyze the addition of CoA to the long-chain polyunsaturated bonds of AA and AdA, respectively, thereby promoting the esterification of PUFAs to membrane PLs.^29,30^ PEs containing acylated AA or AdA (AA/AdA-CoA) can be subsequently oxidized by LOXs to form PL hydroperoxides (PE-AA/AdA-OOH), eventually leading to the membrane deposition of lipid ROS and driving ferroptosis.^246^ A number of inhibitors that target lipid metabolism have been shown to suppress ferroptosis, including LOX inhibitors, radical-trapping antioxidants (RTAs, which scavenge lipid hydroperoxyl radicals), ACSL4 inhibitors, and deuterated PUFAs/MUFAs (by decreasing PUFA-containing PLs).

#### Inhibiting ferroptosis using LOX inhibitors

LOXs are a class of non-heme iron-containing dioxygenases^247^ that can regulate ferroptosis by mediating PL oxidation.^248^ A total of 6 LOX isoforms (ALOX5, ALOX12, ALOX12B, ALOX15, ALOX15B, and ALOXE3) are expressed in humans, and a total of 7 LOX isoforms (Alox5, Alox12, Alox12b, Alox15, Alox15b, Aloxe3, and Alox12e) are expressed in mice.^249,250^ ALOX5/12 inhibitors, including AA-861,^31^ cinnamyl-3,4-dihydroxy-alpha-cyanocinnamate (CDC),^251,252^ the pan-LOX inhibitor nordihydroguaiaretic acid (NDGA),^253^ and BWA4C,^248^ have been either shown or suggested to suppress ferroptosis. In an effort to re-evaluate the selectivity profile of CDC for LOXs, Pergola et al. identified CDC as an ALOX5-specific antagonist with an IC_50_ value in the low nanomolar range (9–25 nM) measured in cell-free assays.^251^ Zileuton, another widely used ALOX5-selective inhibitor, was the first FDA-approved orally administered drug for treating asthma. Zileuton was also shown to exert a neuroprotective effect by blocking ALOX5-mediated glutamate toxicity and ferroptosis in HT22 cells (a mouse hippocampal cell line).^254^ With respect to ALOX12, ML355 is a potent and selective inhibitor^255^ that has been shown to reduce ALOX12-induced thrombosis and protect human pancreatic islets from ALOX12-triggered inflammatory injury.^256,257^ Recently, Zhang et al. reported that the compound IMA-1, which disrupts the interaction between ALOX12 and ACC1 (acetyl-CoA carboxylase 1), can reduce NASH progression in mice and *Cynomolgus* macaques,^258^ suggesting that the ALOX12-ACC1 complex may be a clinically viable target for the management of NASH.

ALOX15 was recently reported to play an essential role in exacerbating I/R-induced cerebral injury^259^ and ischemia-induced myocardial injury^260^ by driving the peroxidation of PL-PUFAs and inducing ferroptosis, providing compelling evidence supporting the viability of targeting ALOX15 to treat I/R-induced injury in these tissues. Interestingly, Walters et al. functionally confirmed that ALOX15 is involved in inducing oxidative stress in human spermatozoa, supporting the notion of increased ALOX15 expression in sperm cells^261^ and suggesting that ALOX15 may be a potential target for the treatment of oxidative stress‒induced male infertility.^261^ Indeed, the ALOX15-selective inhibitor PD146176 was shown to protect against oxidative damage by decreasing the production of membrane PL peroxidation and 4-hydroxynonenal (4-HNE) in spermatozoa.^261^ Moreover, the ALOX15-specific inhibitor ML351 was recently shown to reduce ferroptosis in cardiac I/R-induced injury.^262^

Taken together, various LOX inhibitors have been shown to effectively inhibit ferroptosis. In addition, the enzyme POR (cytochrome P450 oxidoreductase) was recently shown to promote PL peroxidation in a LOX-independent manner,^263^ suggesting that the regulation of enzymes other than LOXs may also contribute to lipid peroxidation by mediating the oxygenation of membrane PLs.

#### Suppressing ferroptosis using radical-trapping antioxidants (RTAs)

One of the principal features of ferroptosis, namely PL peroxidation initiated by the formation and propagation of lipid radicals, can be blocked by RTAs, a class of lipid chain‒breaking antioxidants. Among the various RTAs identified to date, the now-widely used ferrostatin-1 (Fer-1) was originally identified as a potent ferroptosis-specific inhibitor in erastin- and RSL3-treated HT-1080 cells.^1^ By scavenging free radicals, Fer-1 reduces ferroptosis-induced tissue injury and prolongs survival in mouse models of disease.^3,47,64^ However, Fer-1 has not been pursued in drug development, given its metabolic instability and poor pharmacokinetics. Currently, Fer-1 is used as a research tool for studying various ferroptosis-related processes. Compared to Fer-1, second-generation (SRS11-92) and third-generation (SRS16-86) RTAs have significantly higher activity but still contain an ester moiety.^47,64^ To develop an optimized set of RTAs, Devisscher et al. developed a series of compounds in which the ester was replaced with a sulfonamide, providing improved water solubility and better metabolic and kinetic properties.^264^ For example, they found that compound UAMC-3203 was more stable and more potent than Fer-1 (with a significantly lower IC_50_ compared to Fer-1).^264^ After intravenous injection, the terminal plasma half-life of UAMC-3203 is ~3 and 5 h in mice and rats, respectively,^83^ suggesting that this compound has relatively good pharmacokinetics.

Liproxstatin-1 (Lip-1), a spiroquinoxalinamine derivative, was identified by high-throughput screening and shown to inhibit ferroptosis in vivo with better pharmacological properties than Fer-1.^5^ However, Lip-1 also potently inhibits the enzymatic activity of CYP2D6 (with an IC_50_ of 4.1 μM), a member of the cytochrome P450 superfamily of drug-oxidizing enzymes, indicating that Lip-1 may not be suitable for use in clinical trials.^5^

In addition to inhibiting ferroptosis, potent RTAs such as Fer-1 and Lip-1 can insert into membrane PLs via arylamines. Notably, the catalytic activity of both Fer-1 and Lip-1 requires a relatively high temperature.^265^ Using three different cellular models of ferroptosis, Zilka et al. found that tetrahydronapthyridinols (THNs) inhibit ferroptosis induced by RSL3, cystine deprivation, and GPX4 inactivation.^265^ Compared to both Fer-1 and Lip-1, lipophilic THNs have similar potency, while hydrophilic THNs are ineffective at inhibiting ferroptosis.^265^

Diarylamines are RTAs commonly used to reduce the autoxidation of petroleum-derived products. The efficacy of this class of RTAs depends largely on the kinetics and stability of the rapid hydrogen atom transfer to one-electron oxidation by peroxidic species.^266^ Two diarylamine derivatives—phenothiazine and phenoxazine—have been shown to suppress ferroptosis in mouse embryonic fibroblasts.^267^ Interestingly, each phenoxazine molecule can trap >2 peroxyl radicals at moderate temperatures, suggesting new strategies for the development of more potent RTA-based ferroptosis inhibitors. Indeed, Mishima et al. showed that promethazine is more potent at protecting kidney function compared to Fer-1(ref. ^268^), suggesting that promethazine may be a viable tool for studying the role of ferroptosis in various disease models. Notably, edaravone—an RTA clinically approved for treating acute ischemic stroke and amyotrophic lateral sclerosis^135^—has been shown to protect against ferroptosis under various pathological conditions.^269^ In addition, Zilka et al. found that copper(II)-diacetyl-bis(*N*^4^-methylthiosemicarbazone) (CuATSM), a candidate drug for treating ALS and PD, suppresses ferroptosis via its RTA activity.^270^ Recently, a group of reduced forms of vitamin K (VKH_2_), including menaquinone and phylloquinone, was shown to have potent anti-ferroptosis properties,^21^ providing new mechanistic insights into the anti-ferroptotic activity of alternative RTAs.

Recently, Wu et al.^271^ and Barayeu et al.^272^ independently reported that endogenous sulfane sulfur (S^0^) species/hydropersulfides (H_2_S or RSSH) are potent RTAs. By supplying sulfur for S^0^ biosynthesis, cysteine can inhibit ferroptosis via a GPX4-independent mechanism.^272^ Genetic manipulation of enzymes involved in S^0^ biosynthesis clearly implicate S^0^ as playing a role in regulating ferroptosis by scavenging ROS and suppressing lipid peroxides.^272^ Both endogenous and exogenous S^0^ have been shown to provide cellular protection against ferroptosis.^271,272^ Together, these results suggest that regulating ferroptosis by targeting S^0^ warrants further clinical study.

#### Reducing ferroptosis using ACSL4 inhibitors

ACSL4 is a unique and important isozyme involved in the metabolism of PL-PUFAs such as AA, and deleting or inhibiting ACSL4 blocks ferroptosis by preventing the incorporation of PL-PUFAs into cell membranes.^15,30^ Thiazolidinediones (TZDs) such as rosiglitazone, pioglitazone, and troglitazone were originally identified as agonists of peroxisome proliferator-activated receptor γ (PPARγ) and are approved to treat adult type 2 diabetes. Interestingly, TZDs showed effects on suppressing ferroptosis by selectively inhibiting ACSL4^15,273^ in breast cancer cell lines and reduce mortality due to acute renal failure in kidney-specific *Gpx4* knockout mice.^15^

#### Modulators of lipid composition

Similar to ACSL4 inhibitors, deuterated PUFAs and MUFAs can also inhibit ACSL4 by decreasing PL-PUFAs. PUFAs deuterated at the bis-allylic position (D-PUFAs) have been shown to inhibit ferroptosis in cells and animal models of PD and Friedreich’s ataxia.^31,274–276^ Notably, exogenous lipid supplementation can modulate both apoptosis and ferroptosis.^277^ Magtanong et al. showed that exogenous MUFAs can also specifically reduce the accumulation of lipid ROS in the plasma membrane and can displace PUFAs from their cellular location, suppressing ferroptosis in an ACSL3-dependent manner.^277^

Molecules that affect the composition of intracellular lipids may also regulate ferroptosis. For example, sulfosuccinimidyl oleate (SSO), which irreversibly inhibits the fatty acid receptor CD36, has been shown to inhibit ferroptosis in CD8^+^ T cells by blocking the uptake of oxidized lipids.^278^ Moreover, inhibiting stearoyl-CoA desaturase-1 (SCD1) with CVT-11127 or inhibiting acetyl-CoA carboxylase (ACC) with CP-640186 can affect the production of MUFAs, reducing the growth of lung cancer cells.^279^ Thus, Batchuluun et al. proposed that other potent ACC inhibitors should be examined for their ability to affect ferroptosis.^280^

Recently, Minami et al. found that loss of *CDKN2A* expression remodels the lipidome of glioblastoma cells, and patient-derived *CDKN2A*-deficient glioblastoma cells have higher levels of lipid peroxidation, thereby sensitizing the cells to ferroptosis in response to GPX4 inhibition.^281^ These results suggest potential new therapeutic strategies in which targeting cellular lipidome remodeling can induce ferroptosis in glioblastoma cells, particularly in cells lacking *CDKN2A* expression.

#### The mevalonate pathway

The mevalonate pathway regulates ferroptosis by altering GPX4 production by affecting the maturation of selenocysteine tRNA.^108^ The small molecule FIN56 potently induces ferroptosis via two mechanisms, namely by reducing GPX4 levels and by decreasing CoQ_10_ activity by targeting the enzyme SQS, which acts downstream of HMG-CoA (3-hydroxy-3-methylglutaryl-CoA) reductase in the mevalonate pathway.^212,213^

Statins are well-known inhibitors of HMG-CoA reductase (HMGCR) and are commonly used as cholesterol-lowering medications to prevent and/or treat atherosclerosis. Statins have also been shown to increase FIN56-induced cellular ferroptosis^212^ and selectively kill cancer cells in a high-mesenchymal state.^40^ However, the concentration of statins needed to increase ferroptosis in cancer cells is 100–500 times the recommended dose for decreasing cholesterol, and high-dose statins often cause severe side effects such as myalgia, rhabdomyolysis, and hepatotoxicity.^282,283^ To mitigate these side effects, it may be possible to deliver the statins in nanoparticles, leading to a high concentration specifically in tumor cells.^284^

### Additional strategies for targeting ferroptosis

Although numerous ferroptosis-targeting agents have been studied, their properties often make them poorly suited for use as therapeutic compounds; indeed, their low solubility, high metabolic clearance, low cellular permeability, and systemic toxicity limit their clinical development. Therefore, multi-target ferroptosis modulators and nanomaterial technologies have been investigated in an attempt to target ferroptosis more effectively in vivo.

#### Multi-target ferroptosis modulators

Artemisinins, which are derived from sweet wormwood (*Artemisia annua*) extracts, are commonly used as effective antimalarial drugs. The semi-synthetic derivative and major active metabolite of artemisinin, dihydroartemisinin (DHA), has been shown to increase ferroptosis in lung cancer cells by inactivating the PRIM2/SLC7A11 axis,^285^ as well as in head and neck cancers^286^ and glioblastoma cell lines^287^ by inhibiting GPX4. In addition, DHA plays a role in ferritinophagy, thereby inducing free iron‒induced ferroptosis.^288^

Glycyrrhizin, a natural antioxidant extracted from the glycyrrhiza root,^289^ was shown to have protective effects on the liver via its antioxidative, anti-inflammatory, antifibrotic, and antiviral properties.^195,290,291^ Wang et al. showed that glycyrrhizin can prevent ferroptosis in cells and in an animal model of acute liver failure by regulating iron levels, the GSH/GPX4 pathway, and the NRF2/HO-1/HMGB1 pathway.^292^

The flavonoid baicalein (in Chinese: Huangqin) has long been used as a popular antibacterial and antiviral agent.^293^ Near the end of the last century, Afanas’ev et al. reported that baicalein exerts its protective role against oxidative damage by inhibiting the iron-catalyzed Fenton reaction.^294^ In addition, baicalein is a specific antagonist of ALOX12 and was shown to protect against I/R-induced heart injury in mice.^295^ Subsequently, Leyen et al. proposed that baicalein may also protect against I/R-induced brain injury by inhibiting ALOX12/15 (ref. ^296^). Recently, baicalein was shown to prevent ferroptosis in erastin-treated PANC1 cells (a human pancreatic cancer cell line) by reducing ferrous iron, inhibiting GSH consumption, and inhibiting GPX4 degradation, thereby suppressing lipid peroxidation.^297^ These results suggest that baicalein may prevent ferroptosis-associated tissue damage.

#### Targeting ferroptosis using nanoparticles

Compared to small-molecule compounds, ferroptosis-inducing nanoparticles have been suggested to have a better preclinical profile. Importantly, nanoparticles can be loaded with chemotherapeutic drugs or tumor-selective molecules, making them ideally suited for developing nanoparticle-based cancer therapies as well as combination therapies.

**Fig. 6:**
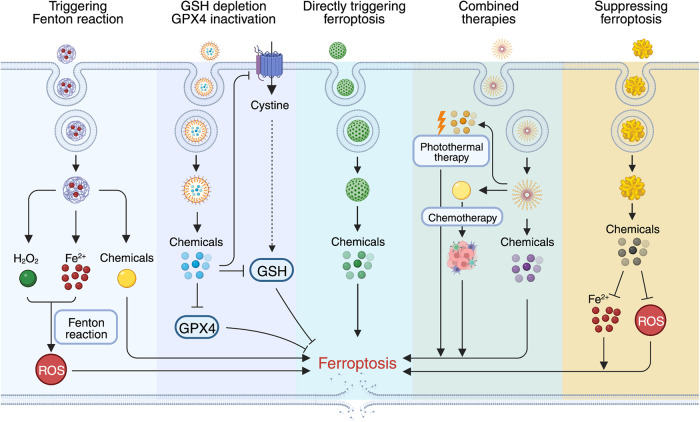
Five strategies for developing nanoparticles to target ferroptosis. Five principal strategies have been used to design nanoparticles to modulate ferroptosis. In all five strategies, nanoparticles contain compounds that either directly modulate ferroptosis or affect signaling pathways that modulate ferroptosis. GSH, glutathione; GPX4, glutathione peroxidase 4; ROS, reactive oxygen species. Created with BioRender.com

Many nanoparticles have been designed to increase ferroptosis by triggering the Fenton reaction in targeted tumor cells (Fig. 6). For instance, the magnetic nanoparticle FeGd-HN@Pt@LF/RGD2,^298^ the PEGylated single-atom Fe-containing nanocatalyst PSAF,^299^ the nanoprobes UCNP@GA-Fe^III^^300^ and UCNP@LP(Azo-CA4),^301^ and the nano-platforms HA@MOF^302^ and PB@FePt-Ha-g-PEG^303^ can effectively deliver Fe^2+^ to cancer cells in order to accelerate the Fenton reaction and produce lethal amounts of ROS, specifically triggering ferroptosis. Interestingly, Zhang et al. found that the FePt@MoS_2_ nanocomposite efficiently induces ferroptosis by releasing >30% of loaded Fe^2+^ into the tumor microenvironment within 72 h.^304^ In addition, GOD-Fe_3_O_4_@DMSNs, in which the nanoparticle is loaded with a natural glucose oxidase, has been shown to exhaust glucose in tumor cells and generate excessive levels of H_2_O_2_, thereby accelerating the Fenton reaction catalyzed by Fe_3_O_4_(ref. ^305^). In the relatively acidic tumor microenvironment, several nanoparticles can be reduced in order to release two molecules such as iron and doxorubicin.^306,307^ For example, FePt-NP2 can deliver iron oxide in order to enhance the chemotherapeutic effects of cisplatin specifically in cancer cells.^308^ Moreover, Shen et al. designed FeGd-HN@Pt@LF/RGD2, a cisplatin-loaded Fe_3_O_4_/Gd_2_O_3_ hybrid nanoparticle conjugated to lactoferrin (LF) and an RGD (Arg-Gly-Asp) dimer in order to potently induce ferroptosis specifically in brain tumor cells.^298^ Thanks to its small size (6.6 nm) and LF receptor‒mediated transcytosis, FeGd-HN@Pt@LF/RGD2 nanoparticles can cross the BBB and deliver cisplatin together with reactants (e.g., Fe^2+^, Fe^3+^, and H_2_O_2_) to brain tumor cells, significantly slowing the growth of orthotopic brain tumors.^298^

An alternative nanoparticle-based strategy for triggering ferroptosis is to deplete GSH and/or inhibit GPX4 (Fig. 6). For example, arginine-rich manganese silicate nanobubbles have been shown to activate ferroptosis by depleting GSH and subsequently inactivating GPX4.^309^ SRF@Fe^III^TA is a rationally designed nanoparticle spontaneously formed by a Fe^3+^ and tannic acid (TA) network‒like corona onto a nanocore containing the kinase inhibitor sorafenib (SRF). In the acidic lysosomal environment, SRF@Fe^III^TA releases SRF, thereby inhibiting the production of GSH and inducing ferroptosis.^310^ Another SRF-based nanoparticle, FaPEG-MnMSN@SFB, has been validated to effectively activate ferroptosis and suppress tumor growth via the dual GSH-depleting effects of MnMSN (manganese-loaded mesoporous silica nanozyme) and SRF.^179^ Moreover, SRF@MPDA-SPIO nanoparticles were generated using SRF and ultrasmall superparamagnetic iron oxide (SPIO) nanoparticles, making the particles visible on MRI and potently inducing ferroptosis by supplying iron.^311^ In addition, the biomimetic magnetic nanoparticle Fe_3_O_4_-SAS@PLT was generated using SAS-loaded mesoporous magnetic nanoparticles (Fe_3_O_4_) and a platelet (PLT) membrane camouflage, thereby activating ferroptosis by blocking the system X_c_^−^ pathway.^174^ Notably, Fe_3_O_4_-SAS@PLT‒induced ferroptosis significantly increased PD-1 (programmed cell death 1)‒based cancer immunotherapy and long-term tumor elimination in mice transplanted with 4T1 metastatic breast cancer cells.^174^ Recently, Li et al. reported the development of the multifunctional nanosheet system Au/Cu-TCPP(Fe)@RSL3-PEG-iRGD, which triggers ferroptosis in tumor cells by simultaneously blocking the GSH/GPX4 and FSP1/CoQ_10_H_2_ pathways via its nanocatalytic activity, as well as synergizing with RSL3 to further inactivate GPX4 in tumor cells.^312^

Interestingly, nanoparticles can also be designed to directly deliver molecules that trigger ferroptosis (Fig. 6). For example, αMSH-PEG-C’ are ultrasmall (<10 nm in diameter) PEG-coated silica nanoparticles conjugated to MC1-R (melanocortin-1 receptor) in order to target alpha melanocyte‒stimulating hormone (αMSH) peptides; this nanoparticle was then shown to activate ferroptosis in a variety of starved cancer cells, as well as in mice xenografted with 786-O and HT-1080 cells.^313^ In addition, MON-p53, an encapsulated metal-organic network (MON) containing a plasmid expressing the tumor suppressor p53, is designed to treat cancer by switching apoptosis to ferroptosis, and long-term treatment (i.e., for 75 days) was shown to have potent anticancer activity in tumor-bearing mice.^314^ Similarly, RSL3 micelles—nanoparticles composed of an AA-conjugated amphiphilic copolymer encapsuling RSL3—have been shown to increase the efficacy of RSL3 in vivo, providing a novel approach to overcome multidrug resistance and improve cancer treatment.^315^ Moreover, a nanodrug consisting of nanoparticle ferritin‒bound erastin and rapamycin (NFER) with significantly improved drug-like properties, was proposed to increase ferroptosis by decreasing GPX4 activity as well as reducing lipid peroxidation levels.^316^ Interestingly, Sal-AuNP, a gold nanoparticle loaded with salinomycin, has been shown to induce ferroptosis in breast cancer stem cells by causing oxidative stress, mitochondrial dysfunction, and lipid oxidation.^317^ Recently, a hypercarbon-centered gold(I) cluster prodrug was engineered and then shown to accelerate ferroptosis in EJ cells (a human bladder cancer cell line) by inhibiting thioredoxin reductase (TrxR) activity.^318^

To maximize the efficacy of cancer therapeutics, nanoparticle-based combination therapies have been widely studied in various preclinical models. For example, CSO-SS-Cy7-Hex/SPION/Srfn^319^ and SPFeN^320^ have been studied as photothermal ferrotherapies for use in cancer, combining photosensitizers with ferroptosis-inducing molecules that can exert synergistic anticancer effects. Another photosensitizer—chlorin e6 (Ce6)—has been used in several delivery systems, including Ce6-erastin nanoparticles,^321^ SRF@Hb-Ce6,^322^ Ce6@MOF,^323^ and HAS-Ce6-IrO_2_.^324^ Liu et al. reported excellent chemodynamic/photodynamic synergy using mCMSN, a biodegradable cancer cell membrane‒coated mesoporous copper/manganese silicate nanosphere that increases ferroptosis via laser irradiation‒triggered ROS production and the GSH-activated Fenton reaction.^325^ To achieve a synergistic ferroptosis and immunomodulatory effect in treating cancer, Zhang et al. engineered a biomimetic magnetosome (Pa-M/Ti-NC) consisting of an Fe_3_O_4_ magnetic nanocluster (NC) as the core, pre-engineered leukocyte membranes loaded with TGF-β inhibitor (Ti), and a PD-1 antibody (Pa) anchored to the membrane surface;^326^ they found that this magnetosome potently promoted ferroptosis in cancer cells via cooperative activity between the Pa and Ti components, causing reprogramming of the immunogenic microenvironment. Interestingly, NMIL-100@Gox@C was designed to activate ferroptosis and induce cancer starvation;^327^ specifically, the glucose oxidase (Gox) enzyme catalyzes glucose to generate sufficient levels of H_2_O_2_ in order to induce ferroptosis, simultaneously causing starvation of the cancer cells via the Gox-mediated consumption of glucose.^327^ Yang et al. developed an HLCaP nanoreactor that encapsulates lipoxidase and hemin in biodegradable PLGA (poly(lactic-co-glycolic acid)), which releases its contents in the acidic tumor microenvironment, synergistically inducing ferroptosis using the PUFA substrates produced in the tumor debris using radiofrequency ablation;^328^ importantly, these so-called tumor debris‒fueled nanoreactors have been shown to prevent tumor recurrence and metastasis.^328^ Similarly, the mesoporous carbon nanoparticle FeCO-Dox@MCN is designed to exploit the combined effects of chemotherapy, photothermal therapy, and gas therapy in order to increase carbon monoxide‒induced ferroptosis.^329^

By contrast, relatively few studies have focused on ferroptosis-suppressing nanoparticles (Fig. 6). Here, we discuss two nanoparticles that have been reported to inhibit ferroptosis. The first is a carboxyl-modified polystyrene nanoparticle (CPS) that effectively inhibits ferroptosis by reducing cellular ROS mediated by TFEB (transcription factor EB).^330^ The second is DEF-HCC-PEG, a deferoxamine-binding nanoparticle that protects cells against both ferroptosis and senescence.^331^ Importantly, ferroptosis is a rapidly developing field, with new mechanisms underlying ferroptosis being identified; thus, additional studies are clearly helpful for accelerating the development of clinical applications using nanoparticles to target ferroptosis.

### Conclusions and future perspectives

Over the past decade, a rapidly growing body of evidence has shown that ferroptosis plays a key role in a wide range of diseases and conditions, including cancer, neurodegenerative disease, tissue/organ injury, inflammation, and inflammatory diseases. Despite many obstacles along the way, it is now widely accepted that targeting ferroptosis holds great promise for promoting the development and clinical translation of ferroptosis-based therapeutic strategies.

## Challenges and opportunities

To date, nearly all in vivo ferroptosis-related studies were based on preclinical animal models, and several challenges limit their translation to clinical practice.

As discussed in this review, numerous molecules—including experimental agents, naturally derived compounds, nanoparticles, and clinically approved drugs—have been shown to modulate ferroptosis, revealing promising new strategies for the development of ferroptosis-based therapies. However, with respect to small molecule compounds, only ~15% of proteins in the human proteome are estimated to be druggable.^332^ In addition, poor pharmacokinetics remains a major bottleneck for the further development of both ferroptosis antagonists such as Fer-1 and Lip-1 and ferroptosis agonists such as RSL3 and ML210. To develop novel bioactive ferroptosis-targeted drug candidates, researchers have studied natural products derived from microorganisms and plants, particularly traditional herbs. Furthermore, high-throughput screening, artificial intelligence, and other new technologies may also be used to identify more effective drug candidates to target ferroptosis.

Another challenge to ferroptosis-based drug discovery is the rational design of combination strategies to treat various ferroptosis-related diseases. It is generally well accepted that ferroptosis plays a pathogenic role in various diseases. In addition, many existing therapies can trigger apoptosis or other types of cell death. For conditions such as cancer, tissue injury, and neurodegenerative disease, various forms of cell death can occur simultaneously. Therefore, developing ferroptosis modulators that can act synergistically with existing therapies may serve as a promising strategy for treating these diseases, particularly drug-resistant cancer. Combining ferroptosis agonists with other anticancer therapies may also increase ferroptosis, as well as other types of cell death, thereby eradicating cancer more effectively.

### Novel technologies for developing ferroptosis-targeting agents

In addition to conventional ferroptosis inhibitors and inducers, new technologies such as proteolysis-targeting chimeras (PROTACs), RNA-based therapies, gene editing, and peptide and protein drugs have been applied to develop ferroptosis-targeting drugs.

#### Targeted protein degradation

Targeted protein degradation (TPD) is a new and challenging therapeutic modality that can degrade “undrugged” targets and other difficult protein targets such as proteins with a broad and shallow active pocket and/or “smooth” surfaces. Three major classifications of protein degraders have been developed, including PROTACs, monomeric targeted protein degraders, and molecular glues (MGs). PROTACs are heterobifunctional small molecules consisting of two ligands connected via a linker. The ligands are designed to bind to an E3 ubiquitin ligase and a target of interest. This chemically induced close proximity between the protein and E3 ligase results in the target protein’s ubiquitylation and subsequent degradation via the ubiquitin-proteasome system (UPS). Recently, TPD has gained considerable attention due to encouraging results obtained from clinical trials involving ARV-110 and ARV-471, two PROTACs that target and degrade the androgen receptor (AR) and estrogen receptor (ER), respectively. Recently, several groups have attempted to develop PROTAC-based ferroptosis modulators. For example, Luo et al. developed the PROTAC dGPX4 to target GPX4 and showed in vivo that it potently induces ferroptosis selectively in cancer cells with no apparent side effects,^333^ providing a promising novel strategy for the development of clinically applicable GPX4-targeted drugs. Similar to PROTACs, photodegradation-targeting chimeras (PDTACs),^334^ lysosome-targeting chimeras (LYTACs),^335^ and autophagy-targeting chimeras (AUTACs)^336^ have also been developed for use in TPD-based therapies. For example, Liu et al. reported the targeted photodegradation of GPX4 using a PDTAC conjugated with the photosensitizer verteporfin upon red-light irradiation, and showed that this PDTAC can induce immunogenic ferroptosis and may improve the efficacy of cancer immunotherapy.^334^

Unlike PROTACs, monomeric targeted protein degraders are designed to either directly bind to their target protein or induce their target protein’s degradation. Therefore, monomeric degraders have a comparatively small molecular weight and can easily cross the BBB.^337^ Fulvestrant, a selective ER degrader, has been approved for treating ER-positive breast cancer, and some monomeric degraders have been tested in clinical trials.^338^ MGs are relatively simple small molecules that increase the interaction between E3 ligase and the protein substrate in order to induce the UPS-induced degradation of target proteins.^339^ Thanks to their low molecular weight, MGs are believed to have more favorable pharmacokinetics than other compounds. Thalidomide was developed as a MG to bring various targets in proximity to the E3 ubiquitin ligase CUL4-RBX1-DDB1-CRBN for degradation.^340^ Lenalidomide, a thalidomide derivative, has been approved to treat multiple myeloma by inducing the degradation of oncoproteins. Thus, monomeric degraders and MGs represent promising new strategies for targeted protein degradation. However, to the best of our knowledge no reports have yet been published regarding the use of monomeric targeted protein degraders or MG-based degraders to target ferroptosis-related proteins.

#### Artificial intelligence (AI)‒based drug discovery strategies

AI, which was first defined by John McCarthy back in 1956 as the “science and engineering of making intelligent machines”, primarily involves computer science, mathematics, and neuroscience, but can also be used for drug discovery.^341^ Given that the traditional process of drug discovery is time-consuming and labor-intensive, it is now widely accepted that AI—particularly machine learning—can significantly increase prediction accuracy and accelerate the drug discovery process. Deep learning, which combines machine learning and AI, can serve as a particularly cost-effective platform for discovering new drugs, as it directly enables rapid machine-based decision-making using an artificial neural network. Today, AI-based technologies have been applied to virtually every step in the drug discovery process, including target identification, drug design, drug screening, chemical synthesis, and prediction of the drug’s properties and mode of action.^341,342^ In addition, AI can be used to quickly identify lead compounds extracted from plants and microorganisms.^343^ Recently, AI was successfully used in cancer research and precision medicine.^344^ With respect to ferroptosis, Wu et al. used a combination of bioinformatics, network pharmacology, and AI to show that ferroptosis and the TGF-β signaling pathway may contribute to the protective effects of celastrol, a plant-derived triterpene, against type 2 diabetes.^345^ It is therefore reasonable to speculate that AI will significantly accelerate the discovery of new ferroptosis-targeted drugs.

#### RNA-based therapies

In addition to protein targets, a new line in the field of drug discovery focuses on RNA-based therapeutics, particularly for the management of genetic diseases. RNA-based therapeutics now include the use of messenger RNA (mRNA), RNAi, single-stranded antisense oligonucleotides, aptamers, ribozymes, and CRISPR-Cas endonuclease‒mediated gene editing. For example, mRNA-based therapies have been successfully used to develop personalized cancer vaccines,^346^ as well as vaccines against infectious diseases.^347^ With respect to RNAi, four RNAi-based therapies have been approved for use in patients, including patisiran for hereditary transthyretin amyloidosis, givosiran for acute hepatic porphyria, lumasiran for primary hyperoxaluria type 1, and inclisiran for heterozygous familial hypercholesterolemia and atherosclerotic cardiovascular disease. These four approved RNAi-based compounds are based on small-interfering RNA (siRNA) technology, which decrease the level of specific mRNAs in order to reduce the corresponding protein levels. Currently, >50 clinical trials are investigating siRNA-based compounds. Unfortunately, however, no clinical trials are investigating ferroptosis-targeting agents using RNA-based technologies. Nevertheless, although the inability to precisely deliver RNA-based tools remains a major barrier to developing RNA therapies, new RNA-based modalities—particularly CRISPR/Cas9-based gene editing—will likely gain considerable traction in the near future.

#### Peptide-based drugs and biologicals

Peptide-based drugs represent a unique pharmaceutical niche between small-molecule compounds and biologicals. Compared to chemical compounds, peptide-based drugs are more efficient, safer, and better tolerated. This category of drugs has several other advantages as well, including high selectivity and reduced tissue accumulation. Biological drugs such as monoclonal antibodies bind with high specificity to their target proteins. Recently, bispecific antibodies—which simultaneously target multiple antigens and/or epitopes using two specific antibodies—have attracted considerable attention and may represent the next generation of antibody-targeted T cell‒based cancer immunotherapies. Furthermore, antibody-drug conjugates have high specificity and are highly efficient at delivering toxic small molecules in order to specifically target cancer cells.

## Conclusions

Ferroptosis is now generally accepted as playing a key pathogenic role in a wide range of diseases and conditions. As summarized in this review, a large amount of compelling evidence supports the notion that targeting ferroptosis can provide promising new options for the treatment of many ferroptosis-related diseases. As our understanding of ferroptosis-regulating pathways—including iron metabolism, lipid metabolism, and oxidative-reductive pathways—increases to reveal new druggable targets, ferroptosis modulators are likely to provide new opportunities to develop treatments for many currently incurable diseases. Although further studies are clearly needed, the novel technologies summarized in this review will facilitate the development of safer, more effective ferroptosis-targeted drugs.
